# Synergic interaction between ritodrine and magnesium sulfate on the occurrence of critical neonatal hyperkalemia: A Japanese nationwide retrospective cohort study

**DOI:** 10.1038/s41598-020-64687-w

**Published:** 2020-05-08

**Authors:** Yukari Yada, Akihide Ohkuchi, Katsufumi Otsuki, Keiji Goishi, Mari Takahashi, Naohiro Yonemoto, Shigeru Saito, Satoshi Kusuda, The Survey Group Studying the Effects of Tocolytic Agents on Neonatal Adverse Events in Japan Society of Perinatal and Neonatal Medicine, Hajime Ota, Kiyotaka Kosugiyama, Kazuhiko Okuyama, Masato Mizushima, Hideaki Negishi, Shinichi Koshida, Mayumi Kasai, Motonari Okabe, Akira Sato, Hiroyuki Adachi, Michio Banzai, Kazuhiro Akaba, Rika Suzuki, Naohisa Ishibashi, Takashi Watanabe, Yoshio Kasuga, Takashi Kameda, Toru Fujiu, Takeshi Takagi, Kenichi Maruyama, Masahiko Higashino, Tomomi Naito, Yoshimasa Kamei, Tetsuya Kunikata, Yoshinori Iitsuka, Harumi Otsuka, Yuka Yamamoto, Mie Yamada, Masaki Daigo, Hironobu Hyodo, Ayumi Sato, Noriko Kataoka, Satoko Yamanaka, Aya Okahashi, Yuki Kojima, Shigenori Kabashima, Yoshie Nakamura, Rina Okuno, Seiko Hirose, Koichi Sugahara, Satsuki Okamoto, Sumiko Hara, Wakako Shima, Takeshi Suzuki, Hideyuki Kagawa, Kenichiro Fujioka, Akiko Kurasaki, Ayako Miura, Isamu Hokuto, Toru Arase, Nobuhiko Taguchi, Kazuki Sekiguchi, Tomoyo Matsuo, Emi Ohnuma, Kana Fujiwara, Miyuki Ogawa, Azusa Uozumi, Noriyuki Yokomichi, Akane Hirose, Mika Okuda, Ayako Fukuyama, Hitoshi Ishimoto, Kanako Mitsuzuka, Shinya Kondo, Miyuki Kitazawa, Norihiko Kikuchi, Yumiko Miyashita, Chiharu Tsutsumi, Shuhei Terada, Shigeru Ohki, Takakazu Kawamura, Masako Yasuda, Yoshiki Soeno, Takumi Kurabayashi, Yoshihisa Nagayama, Satoshi Yoneda, Tomomi Shiga, Seiji Hayashi, Hiroyuki Tsuda, Makoto Oshiro, Takafumi Ushida, Teruyuki Mizutani, Hideyuki Asada, Ryousuke Miura, Ryo Tanaka, Noriko Kato, Yuko Sasaki, Takehiko Yokoyama, Takako Hirooka, Takaharu Yamada, Kaori Maruwaka, Syunsuke Nagara, Satoko Fukaya, Mari Koroki, Taihei Tanaka, Shigehiko Morikawa, Shigeru Honda, Haruki Sassa, Takeshi Sahashi, Hiroko Torii, Tadahiro Yasuo, Nozomi Kuriyama, Juzo Okada, Moe Kano, Noriyoshi Oki, Mieko Inagaki, Yousuke Mizuno, Masayo Fujisaka, Akihiro Takatera, Takeo Mure, Katsuhiko Yoshii, Yasuko Furuichi, Akiko Kanto, On Fukui, Shusaku Hayashi, Hitomi Ono, Eri Fujikawa, Masayuki Someya, Makiko Ikeda, Kentaro Nakanishi, Akiko Yamashita, Haruna Kawaguchi, Ryo Yamamoto, Jun Sasahara, Takeshi Kanagawa, Satoshi Yamamoto, Yosuke Imanishi, Misuzu Yoshida, Eri Yano, Ayumi Murayama, Kazue Morikawa, Natsuko Tabata, Ryosuke Araki, Eriko Iwasaki, Narutaka Mochizuki, Akiko Kobayashi, Akiko Takeda, Akiko Kobayashi, Masaya Hirose, Nao Taguchi, Hiroshi Sato, Kenji Oida, Rie Sakai, Saeko Imai, Reona Shiro, Minami Okudate, Yoko Matsuda, Yoshinobu Nishida, Aya Toyofuku, Shigeto Hara, Hiroko Kurioka, Tomoya Mizunoe, Syouhei Eto, Takahiro Nobuzane, Kousyou Higuchi, Terumi Miwa, Keiko Hasegawa, Yuko Matsubara, Masaaki Ohta, Takafumi Watanabe, Takako Ohmaru-Nakanishi, Kana Kashinoura, Maki Goto, Hiroshi Kanda, Kiyomi Tsukimori, Yasushi Takahata, Makoto Nomiyama, Toshimitsu Takayanagi, Syuichiro Yoshimura, Kouhei Kotera, Hisanobu Fukuda, Hiroko Hiraki, Noriko Nagata, Kazuhisa Nakashima, Junya Miyoshi, Takafumi Obara, Kentaro Kai, Yuichi Furukawa, Satoshi Eto, Tomoko Oishi, Misaki Nakashima, Aya Yamauchi, Yuki Kodama, Takako Ohata, Haruka Arakaki, Kei Miyakoshi, Mariko Hida

**Affiliations:** 1 Department of Pediatrics, Jichi Medical University School of Medicine, Tochigi, Japan; 2 Department of Obstetrics and Gynecology, Jichi Medical University School of Medicine, Tochigi, Japan; 3 Department of Obtetrics and Gynecology, Showa University Koto Toyosu Hospital, Tokyo, Japan; 4 Department of Pediatrics, National Center for Global Health and Medicine, Tokyo, Japan; 5 Japan Society of Perinatal and Neonatal Medicine, Tokyo, Japan; 6 Department of Psychopharmacology, National Center of Neurology and Psychiatry, Tokyo, Japan; 7 Department of Obstetrics and Gynecology, University of Toyama, Toyama, Japan; 8 Department of Pediatrics, Kyorin University, Tokyo, Japan; 9 Teine Keijinkai Hospital, Hokkaido, Japan; 10 Sapporo City General Hospital, Hokkaido, Japan; 11 Japanese Red Cross Hospital Kitami, Hokkaido, Japan; 12 Iwate Prefectural Central Hospital, Iwate, Japan; 13 Akita University, Akita, Japan; 14 Yamagata Saisei Hospital, Yamagata, Japan; 15 Ohara General Hospital, Fukushima, Japan; 16 Haga Red Cross Hospital, Tochigi, Japan; 17 Japanese Red Cross Ashikaga Hospital, Tochigi, Japan; 18 Gunma University, Gunma, Japan; 19 Gunma Children’s Medical Center, Gunma, Japan; 20 Saiseikai Kawaguchi General Hospital, Saitama, Japan; 21 Saitama Medical University Hospital, Saitama, Japan; 22 Chiba Kaihin Municipal Hospital, Chiba, Japan; 23 Juntendo University Urayasu Hospital, Chiba, Japan; 24 San-Ikukai Hospital, Tokyo, Japan; 25 Tokyo Metropolitan Bokutoh Hospital, Tokyo, Japan; 26 NTT Medical Center Tokyo, Tokyo, Japan; 27 International Catholic Hospital, Tokyo, Japan; 28 Nihon University Itabashi Hospital, Tokyo, Japan; 29 Tachikawa Sougo General Hospital, Tokyo, Japan; 30 Fussa Hospital, Tokyo, Japan; 31 Tokyo Adventist Hospital, Tokyo, Japan; 32 Kawasaki Municipal Hospital, Kanagawa, Japan; 33 Kanto Rosai Hospital, Kanagawa, Japan; 34 St. Marianna university School of Medicine, Kanagawa, Japan; 35 Keiyu Hospital, Kanagawa, Japan; 36 Kitasato University, Kanagawa, Japan; 37 Saiseikai Yokohamashi Nanbu Hospital, Kanagawa, Japan; 38 Yokohama City University Hospital, Kanagawa, Japan; 39 St. Marianna University School of Medicine, Yokohama City Seibu Hospital, Kanagawa, Japan; 40 National Hospital Organization Yokohama Medical Center, Kanagawa, Japan; 41 Tokai University, Kanagawa, Japan; 42 Kanagawa Children’s Medical Center, Kanagawa, Japan; 43 Shinonoi General Hospital, Nagano, Japan; 44 Shinshu University, Nagano, Japan; 45 Shizuoka City Shimizu Hospital, Shizuoka, Japan; 46 Seirei Hamamatsu General Hospital, Shizuoka, Japan; 47 Shizuoka Children’s Hospital, Shizuoka, Japan; 48 Nagaoka Red Cross Hospital, Niigata, Japan; 49 Niigata City General Hospital, Niigata, Japan; 50 Gifu University, Gifu, Japan; 51 Okazaki City Hospital, Aichi, Japan; 52 Japanese Red Cross Nagoya Daiichi Hospital, Aichi, Japan; 53 Nagoya University, Aichi, Japan; 54 Japanese Red Cross Nagoya Daini Hospital, Aichi, Japan; 55 Komaki City Hospital, Aichi, Japan; 56 Ichinomiya Municipal Hospital, Aichi, Japan; 57 Kusatsu General Hospital, Shiga, Japan; 58 Japanese Red Cross Kyoto Daiichi Hospital, Kyoto, Japan; 59 Chibune General Hospital, Osaka, Japan; 60 Higashiosaka City Medical Center, Osaka, Japan; 61 Kindai University, Osaka, Japan; 62 Sakai City Medical Center, Osaka, Japan; 63 Osaka Women’s and Children’s Hospital, Osaka, Japan; 64 National Hospital Organization Kobe Medical Center, Hyogo, Japan; 65 Hyogo Prefectural Amagasaki General Medical Center, Hyogo, Japan; 66 Japanese Red Cross Wakayama Medical Center, Wakayama, Japan; 67 Shimane Prefectural Central Hospital, Shimane, Japan; 68 National Hospital Organization Kure Medical Center, Hiroshima, Japan; 69 Chugoku Rosai Hospital, Hiroshima, Japan; 70 Yamaguchi Grand Medical Center, Yamaguchi, Japan; 71 Ehime University, Ehime, Japan; 72 Kochi Health Sciences Center, Kochi, Japan; 73 Hamanomachi Hospital, Fukuoka, Japan; 74 National Hospital Organization Kyushu Medical Center, Fukuoka, Japan; 75 Iizuka Hospital, Fukuoka, Japan; 76 Fukuoka Children’s Hospital, Fukuoka, Japan; 77 National Hospital Organization Saga Hospital, Saga, Japan; 78 Nagasaki Harbor Medical Center, Nagasaki, Japan; 79 Japanese Red Cross Kumamoto Hospital, Kumamoto, Japan; 80 Nakatsu Municipal Hospital, Oita, Japan; 81 Miyazaki Prefectural Nobeoka Hospital, Miyazaki, Japan; 82 University of Miyazaki, Miyazaki, Japan; 83 Okinawa Chubu Hospital, Okinawa, Japan; 84 Keio University Hospital, Tokyo, Japan

**Keywords:** Epidemiology, Risk factors

## Abstract

Our aim was to evaluate the association between ritodrine and magnesium sulfate (MgSO_4_) and the occurrence of neonatal hyperkalemia or hypoglycemia among late preterm infants in a retrospective cohort study. We used a nationwide obstetrical database from 2014. A total of 4,622 live preterm infants born at 32–36 gestational weeks participated. Fourteen risk factors based on both clinical relevance and univariate analysis were adjusted in multivariable logistic regression analyses. Neonatal hyperkalemia and hypoglycemia occurred in 7.6% (284/3,732) and 32.4% (1,458/4,501), respectively. Occurrence of hyperkalemia was associated with concomitant usage of ritodrine and MgSO_4_ compared with no usage (adjusted odds ratio [aOR] 1.53, 95% confidence interval [CI] 1.09–2.15). Occurrence of hypoglycemia was associated with ritodrine alone (aOR 2.58 [CI 2.21–3.01]) and with concomitant usage of ritodrine and MgSO_4_ (aOR 2.59 [CI 2.13–3.15]), compared with no usage, and was associated with long-term usage (≥ 48 hours) of ritodrine and cessation directly before delivery. In conclusion, in late preterm infants, usage of ritodrine together with MgSO_4_ was associated with occurrence of critical neonatal hyperkalemia, and long-term usage of ritodrine and cessation directly before delivery were associated with neonatal hypoglycemia.

## Introduction

The Cause Analysis Committee for Cerebral Palsy of the Japan Council for Quality Health Care (JCQHC) has suggested that of the nearly 1,000 cases of cerebral palsy, some may have occurred in neonates with hypoglycemia and/or hyperkalemia born to mothers receiving either ritodrine or magnesium sulfate (MgSO_4_)^1^. In addition, 14 cases of unexpected neonatal hyperkalemia from mothers using tocolytic agents^2–14^ –especially ritodrine and MgSO_4_ concomitantly^2,3^ – have been independently reported by neonatologists in Japan. At JCQHC request, the Japan Society of Perinatal and Neonatal Medicine (JSPNM) is evaluating the association between these agents and neonatal hyperkalemia or hypoglycemia occurrence. However, to the best of our knowledge, there have been no cohort studies in English suggesting a relationship between ritodrine administration and neonatal hyperkalemia. Regarding MgSO_4_, there has only been one case reported of a very low birth weight infant with hyperkalemia accompanied by hypermagnesemia^15^.

In 2013 the US Food and Drug Administration (FDA) and European Medicines Agency (EMA) recommended against long-term tocolysis ≥48 hours with ritodrine and MgSO_4_^16–18^. Following these restrictions, Kissei Pharmaceutical Co. Ltd. manufacturing ritodrine in Japan, published “A review of EU restrictions on short-acting beta-agonists, and guidelines regarding efficacy and safety of ritodrine hydrochloride (injection and tablet) in Japan” in Dec. 2014^19^. Accordingly, two leading societies for obstetrical decision-making in Japan, the Japan Society of Obstetrics and Gynecology (JSOG) and Japan Association of Obstetricians and Gynecologists (JAOG), have not prohibited long-term tocolysis using ritodrine^20,21^. Therefore, long-term tocolysis using ritodrine has often been performed^22^.

Although MgSO_4_ had been used for seizure prophylaxis in women with preeclampsia or eclampsia^23^, its usage as a tocolytic agent became covered by insurance in Japan in 2006^24^. Generally, maintenance therapy with MgSO_4_ is not recommended in the USA^25^; however, long-term MgSO_4_ tocolysis is widely performed in Japan because the package insert does not prohibit usage at 22–36 gestational weeks^24^. In addition, long-term tocolysis with both ritodrine and MgSO_4_ has been used in Japan when uterine contractions could not be controlled using ritodrine or MgSO_4_ alone^26–29^.

Ritodrine and MgSO_4_ have been used widely as tocolytic agents in Japan^20,21^. Although hyperglycemia and hypokalemia are well-known adverse events of ritodrine in pregnant women^30^, and hypoglycemia is a well-known adverse event in neonates born to mothers receiving ritodrine^30^, whether ritodrine usage in preterm labor is associated with increased risk of neonatal hyperkalemia is unknown. Furthermore, although hyperkalemia has been reported as a rare adverse event of MgSO_4_ in pregnant women^24^, it is unknown whether MgSO_4_ usage in women during preterm labor is also associated with increased neonatal hyperkalemia risk. To our best knowledge, association between ritodrine and/or MgSO_4_ usage and neonatal hyperkalemia occurrence has not been documented in a large cohort study.

We hypothesized that long-term tocolysis with either ritodrine or MgSO_4_ until 36 gestational weeks, or the combination of ritodrine and MgSO_4_ may be associated with increased risks of neonatal hypoglycemia or hyperkalemia in Japan. Moreover, we also hypothesized neonatal hypoglycemia or hyperkalemia may be associated with increased risks of abnormal neurological findings including cerebral palsy. Therefore, our main aim was to investigate hyperkalemia and hypoglycemia incidence in neonates born to mothers receiving either ritodrine or MgSO_4_ therapy for preterm labor. Our secondary aim was to investigate the association between neonatal hypoglycemia and hyperkalemia and later occurrence of abnormal neurological findings including cerebral palsy. Our study focused only on late preterm infants born after 32 gestational weeks. Such preterm infants are usually not severely ill at birth, and tend to be cared for in step down neonatal units or obstetrical wards without close examination, unlike neonatal intensive care units (NICUs).

## Results

### Maternal and infantile characteristics based on presence /absence of hypoglycemia

Hypoglycemia occurred in 32.4% at birth and at <3 hours after delivery in 94.3% of cases. Frequencies of the following were significantly higher in infants with hypoglycemia: Mother with PL/shortened CL/CI (preterm labor/shortened cervical length/cervical incompetency), placenta previa/low-lying placenta, cesarean section, twins/triplets, small-for-gestational-age (SGA) infants, and women with ritodrine alone or the concomitant usage of both ritodrine and MgSO_4_. However, in infants with hypoglycemia, frequencies of women with preterm premature rupture of the membranes (pPROM), and GH/PE/eclampsia/ HELLP/AFLP (gestational hypertension/ preeclampsia/eclampsia/hemolysis, elevated liver enzymes, and low platelets/acute fatty liver of pregnancy) were significantly lower (Table 1).

**Table 1:** Maternal and infantile characteristics in 4,501 infants with data on hypoglycemia who were born at 32–36 gestational weeks.

Characteristics	Non-hypoglycemia	Hypoglycemia	Missing value	*P*-value
	(N = 3,043)	(N = 1,458)		
Maternal characteristics				
Age (yr)	32.5 (29.5–36.5)	33.5 (29.5–37.5)	1	0.079
Nulliparity	1,509 (49.6)	704 (48.3)	0	0.426
Possible maternal risk factors for hypoglycemia				
Obstetrical complications				
**TPL/shortened CL/CI**	1,612 (53.0)	965 (66.2)	0	**<0.001**
**pPROM**	884 (29.1)	290 (19.9)	0	**<0.001**
**GH/PE/eclampsia/HELLP/AFLP**	483 (15.9)	198 (13.6)	0	**0.046**
Placental abruption	93 (3.1)	37 (2.5)	0	0.392
**Placenta previa/Low-lying placenta**	175 (5.8)	114 (7.8)	0	**0.009**
DM	29 (1.0)	16 (1.1)	0	0.634
GDM	159 (5.2)	77 (5.3)	0	0.943
**Cesarean section**	1,755/3,018 (58.2)	1,071/1,432 (74.8)	51	**<0.001**
**MgSO**_**4**_ **usage**	606 (19.9)	362 (24.8)	0	**<0.001**
**Ritodrine usage**	1,371 (45.1)	988 (67.8)	0	**<0.001**
Combination of MgSO_4_ and ritodrine^a^				
Neither MgSO_4_ nor ritodrine	1,486 (48.8)	423 (29.0)	0	**<0.001**
MgSO_4_ alone	186 (6.1)	47 (3.2)		
**Ritodrine alone**	951 (31.3)	673 (46.2)		
**Both MgSO**_**4**_ **and ritodrine**	420 (13.8)	315 (21.6)		
Possible children’s risk factors for hypoglycemia				
Gestational weeks at delivery	35.6 (34.2–36.4)	35.6 (34.2–36.4)	0	0.617
Delivery at <35 wk	1,169 (38.4)	563 (38.6)	0	0.922
**Birthweight (g)**	2,202 (1,916–2,464)	2,150 (1,820–2,432)	0	**<0.001**
**Twins/Triplets**	770 (25.3)	572 (39.2)	0	**<0.001**
Sex: male	1,732/3,042 (56.9)	788 (54.0)	1	0.072
**SGA infants**	342 (11.2)	196 (13.4)	0	**0.035**
Large-for-gestational-age infants	33 (1.1)	18 (1.2)	0	0.654
Apgar score at 1 min <3	93/3,040 (3.1)	43/1,456 (3.0)	5	0.926

Abbreviations: yr, years old; TPL, threatened preterm labor; CL, cervical length; CI, cervical incompetency; pPROM, preterm premature rupture of the membranes; GH, gestational hypertension; PE, preeclampsia; HELLP, hemolysis, elevated liver enzymes, and low platelets; AFLP, acute fatty liver of pregnancy; DM, diabetes mellitus; GDM, gestational diabetes mellitus; wk, gestational weeks; SGA, small-for-gestational-age; min, minute.

This analysis was performed using”Hypoglycemia set”.

Continuous variables are shown as median (interquartile range), and discrete variables are shown as n (%).

The statistical differences between infants with vs. without hypoglycemia were tested using the Mann-Whitney test, Fisher’s exact test, or χ^2^ test.

^a^ The incidence of HG in women with “no usage of either MgSO_4_ or ritodrine (Group 1: G1)”, “MgSO_4_ alone (Group 2: G2)”, “ritodrine alone (Group 3: G3)”, and “both MgSO_4_ and ritodrine (Group 4: G4)” was 22.2, 20.2, 41.4, and 42.9%, respectively. Significant pairs by Bonferroni test were G1 vs. G3, G1 vs. G4, G2 vs. G3, and G2 vs. G4.

### Effects of various risk factors on hypoglycemia occurrence

In multivariable logistic regression analyses using the “Hypoglycemia set”, independent risk factors for hypoglycemia occurrence were cesarean section, SGA infants, and delivery to a mother with ritodrine alone or concomitant usage of ritodrine and MgSO_4_ (Table 2). Interestingly, preterm premature rupture of the membranes (pPROM) was a negative independent risk factor for hypoglycemia occurrence.

**Table 2:** Effects of various risk factors on hypoglycemia occurrence in 4,501 infants born at 32–36 gestational weeks.

Risk factors^a^	Univariate analysis			Multivariable analysis^b^		
	Crude odds ratio (95% CI)		*P*-value	Adjusted odds ratio (95% CI)		*P*-value
Combination of MgSO_4_ and ritodrine						
MgSO_4_ alone vs. no usage	0.89	(0.63–1.24)	0.489	0.81	(0.56–1.16)	0.244
**Ritodrine alone vs. no usage**	2.49	(2.15–2.88)	<0.001	**2.58**	**(2.21–3.01)**	**<0.001**
**Both MgSO**_**4**_ **and ritodrine vs. no usage**	2.64	(2.20–3.16)	<0.001	**2.59**	**(2.13–3.15)**	**<0.001**
Obstetrical complications						
**pPROM**	0.61	(0.52–0.71)	<0.001	**0.72**	**(0.61–0.85)**	**<0.001**
GH/PE/eclampsia/HELLP/AFLP	0.83	(0.70–0.996)	0.045	0.91	(0.74–1.12)	0.355
Placental abruption	0.83	(0.56–1.22)	0.332	0.91	(0.60–1.38)	0.670
Placenta previa/Low lying placenta	1.39	(1.09–1.78)	0.008	0.96	(0.74–1.26)	0.782
DM	1.15	(0.62–2.13)	0.649	1.62	(0.85–3.08)	0.145
GDM	1.01	(0.77–1.34)	0.937	1.14	(0.85–1.54)	0.372
**Cesarean section**	2.14	(1.86–2.46)	<0.001	**1.96**	**(1.67–2.31)**	**<0.001**
Delivery at <35 wk	1.01	(0.89–1.15)	0.898	0.95	(0.82–1.09)	0.646
**Twins/triplets**	1.91	(1.67–2.18)	<0.001	**1.23**	**(1.06–1.44)**	**0.009**
Sex: male	0.89	(0.79–1.01)	0.068	0.91	(0.80–1.04)	0.166
**SGA infants**	1.23	(1.02–1.48)	0.033	**1.35**	**(1.10–1.65)**	**0.004**
Large-for-gestational-age infants	1.14	(0.64–2.03)	0.656	1.58	(0.85–2.94)	0.146
Apgar score at 1 min <3	0.96	(0.67–1.39)	0.846	0.98	(0.66–1.45)	0.917

Abbreviations: CI, confidence interval; MgSO_4_, magnesium sulfate; pPROM, preterm premature rupture of the membranes; GH, gestational hypertension; PE, preeclampsia; HELLP, hemolysis, elevated liver enzymes, and low platelets; AFLP, acute fatty liver of pregnancy; DM, diabetes mellitus; GDM, gestational diabetes mellitus; wk, gestational weeks; SGA, small-for-gestational-age; min, minute.

This analysis was performed using”Hypoglycemia set”.

^a^Risk factors were determined based on both clinical relevance and univariate analysis as follows: combination of MgSO_4_ and ritodrine, obstetrical complications, cesarean section, delivery at <35 wk, twins/triplets, infantile sex, SGA infants, large-for-gestational-age infants, and Apgar score at 1 min <3.

^b^Multivariable analyses were performed using the same risk factors as in univariate analyses. However, birthweight was not used as a risk factor due to the close relationship with gestational weeks. In addition, the obstetric complication of TPL/shortened CL/CI was not used as a risk factor because either ritodrine or MgSO_4_ was commonly used under these conditions. Excluding 57 patients with missing data for 14 variables, a total of 4,444 patients underwent multivariable analysis. Abbreviations: TPL, threatened preterm labor; CL, cervical length; CI, cervical incompetency.

### Maternal and infantile characteristics based on presence/absence of hyperkalemia

Hyperkalemia occurred in 7.6% of cases at birth, and 24.0% at <3 hours, 9.2% at 3–5 hours, 53.0% at 6–23 hours, and 13.8% at ≥24 hours. Frequencies of hyperkalemia were significantly higher in infants born at <35 gestational weeks, with an Apgar score at 1 minute <3, and whose mother used ritodrine and MgSO_4_ concomitantly (Table 3)

**Table 3:** Maternal and infantile characteristics in 3,732 infants with data on hyperkalemia who were born at 32–36 gestational weeks.

Characteristics	Non-hyperkalemia	Hyperkalemia	Missing value	*P*-value
	(N = 3,448)	(N = 284)		
Maternal characteristics				
Age (yr)	33.5 (29.5–36.5)	33.5 (29.8–37.5)	2	0.221
Nulliparity	1,766 (51.2)	161 (56.7)	0	0.084
Possible maternal risk factors for hyperkalemia				
Obstetrical complications				
TPL/shortened CL/CI	1,973 (57.2)	175 (61.6)	0	0.152
pPROM	953 (27.6)	65 (22.9)	0	0.096
GH/PE/eclampsia/HELLP/AFLP	561 (16.3)	54 (19.0)	0	0.244
Placental abruption	119 (3.5)	6 (2.1)	0	0.302
Placenta previa/Low-lying placenta	214 (6.2)	23 (8.1)	0	0.206
DM	38 (1.1)	2 (0.7)	0	0.766
GDM	163 (4.7)	20 (7.0)	0	0.086
Cesarean section	2,229/3,417 (65.2)	191/281 (68.0)	34	0.362
**MgSO**_**4**_ **usage**	770 (22.3)	82 (28.9)	0	**0.015**
**Ritodrine usage**	1,830 (53.1)	174 (61.3)	0	**0.008**
Combination of MgSO_4_ and ritodrine^a^				
neither MgSO_4_ nor ritodrine	1,418 (41.1)	98 (34.5)	0	**0.003**
MgSO_4_ alone	200 (5.8)	12 (4.2)		
Ritodrine alone	1,260 (36.5)	104 (36.6)		
**Both MgSO**_**4**_ **and ritodrine**	570 (16.5)	70 (24.6)		
Possible children’s risk factors for hyperkalemia				
**Gestational weeks at delivery**	35.2 (34.1–36.2)	34.8 (33.4–36.1)	0	**<0.001**
**Delivery at <35 wk**	1,549 (44.9)	154 (54.2)	0	**0.003**
**Birthweight (g)**	2,126 (1,832–2,388)	2,056 (1,814–2,363)	0	**0.042**
Twins/Triplets	1,005 (29.1)	91 (32.0)	0	0.310
Sex: male	1,937/3,447 (56.2)	153 (53.9)	1	0.456
SGA infants	462 (13.4)	34 (12.0)	0	0.585
Large-for-gestational-age infants	33 (1.0)	1 (0.4)	0	0.513
**Apgar score at 1 min** < **3**	112/3,443 (3.3)	18 (6.3)	5	0.011

Abbreviations: yr, years old; GH, gestational hypertension; PE, preeclampsia; TPL, threatened preterm labor; CL, cervical length; CI, cervical incompetency; pPROM, preterm premature rupture of the membranes; GH, gestational hypertension; PE, preeclampsia; HELLP, hemolysis, elevated liver enzymes, and low platelets; AFLP, acute fatty liver of pregnancy; DM, diabetes mellitus; GDM, gestational diabetes mellitus; wk, gestational weeks; SGA, small-for-gestational-age; min, minute.

This analysis was performed using “Hyperkalemia set”.

Continuous variables are shown as median (interquartile range), and discrete variables are shown as n (%).

The statistical differences between infants with hypoglycemia vs. those without were tested using the Mann-Whitney test, Fisher’a exact test, or χ^2^ test.

^a^The incidence of HK in women with “no usage of either MgSO_4_ or ritodrine (Group 1: G1)”, “MgSO_4_ alone (Group 2: G2)”, “ritodrine alone (Group 3: G3)”, and “both MgSO_4_ and ritodrine (Group 4: G4)” was 6.5, 5.7, 7.6, and 10.9%, respectively. Significant pair by Bonferroni test was G1 vs. G4.

### Effects of various risk factors on hyperkalemia occurrence

In multivariable logistic regression analyses using the “Hyperkalemia set”, independent risk factors for hyperkalemia occurrence were delivery at <35 gestational weeks, an Apgar score at 1 minute <3, and delivery to a mother with concomitant usage of ritodrine and MgSO_4_ (Table 4).

**Table 4:** Effects of various risk factors on hyperkalemia occurrence in 3,732 infants who were born at 32–36 gestational weeks.

Risk factors^a^	Univariate analysis			Multivariable analysis^b^		
	Crude odds ratio (95% CI)		*P*-value	Adjusted odds ratio (95% CI)		*P*-value
**Combination of MgSO**_**4**_ **and ritodrine**						
MgSO_4_ alone vs. no usage	0.87	(0.47–1.61)	0.654	0.63	(0.33–1.20)	0.155
Ritodrine alone vs. no usage	1.19	(0.90–1.59)	0.224	1.20	(0.89–1.62)	0.231
**Both MgSO**_**4**_ **and ritodrine vs. no usage**	1.78	(1.29–2.45)	<0.001	**1.53**	**(1.09–2.15)**	**0.015**
**Obstetrical complications**						
pPROM	0.78	(0.58–1.04)	0.085	0.78	(0.57–1.07)	0.118
GH/PE/eclampsia/HELLP/AFLP	1.21	(0.89–1.65)	0.232	1.37	(0.96–1.96)	0.085
Placental abruption	0.60	(0.26–1.38)	0.233	0.46	(0.19–1.09)	0.076
Placenta previa/Low lying placenta	1.33	(0.85–2.09)	0.210	1.24	(0.76–2.02)	0.389
DM	0.64	(0.15–2.65)	0.535	0.85	(0.20–3.56)	0.818
GDM	1.53	(0.94–2.47)	0.085	1.63	(0.998–2.67)	0.051
Cesarean section	1.13	(0.87–1.47)	0.354	0.96	(0.71–1.31)	0.798
**Delivery at <35 wk**	1.45	(1.14–1.85)	0.003	**1.46**	**(1.13–1.88)**	**0.004**
Twins/triplets	1.15	(0.88–1.49)	0.303	1.11	(0.82–1.50)	0.501
Sex: male	0.91	(0.71–1.16)	0.994	0.92	(0.72–1.18)	0.511
SGA infants	0.88	(0.61–1.27)	0.496	0.87	(0.59–1.28)	0.477
Large-for-gestational-age infants	0.37	(0.05–2.68)	0.323	0.35	(0.05–2.61)	0.306
**Apgar score at 1 min** < **3**	2.01	(1.21–3.36)	0.008	**2.21**	**(1.29–3.81)**	**0.004**

Abbreviations: CI, confidence interval; MgSO_4_, magnesium sulfate; pPROM, preterm premature rupture of the membranes; GH, gestational hypertension; PE, preeclampsia; HELLP, hemolysis, elevated liver enzymes, and low platelets; AFLP, acute fatty liver of pregnancy; DM, diabetes mellitus; GDM, gestational diabetes mellitus; wk, gestational weeks; SGA, small-for-gestational-age; min, minute.

This analysis was performed using “Hyperkalemia set”.

^a^Risk factors were determined based on both clinical relevance and univariate analysis as follows: combination of MgSO_4_ and ritodrine, obstetrical complications, cesarean section, delivery at <35 wk, twins/triplets, infantile sex, SGA infants, large-for-gestational-age infants, and Apgar score at 1 min <3.

^b^Multivariable analyses were performed using all the risk factors using the univariate analyses. However, birthweight was not used as a risk factor due to the close relationship with gestational weeks. In addition, the obstetric complication of TPL/shortened CL/CI was not used as a risk factor because either ritodrine or MgSO_4_ was commonly used under these conditions. Excluding 40 patients with missing data for 14 variables, a total of 3,692 patients underwent multivariable analysis. abbreviations: TPL, threatened preterm labor; CL, cervical length; CI, cervical incompetency.

### Maternal and infantile characteristics with the combination of MgSO_4_ and ritodrine

Data from four usage groups – MgSO_4_ alone, ritodrine alone, both MgSO_4_ and ritodrine, and neither MgSO_4_ nor ritodrine – are shown in Supplementary Table S1. MgSO_4_ was the smallest group (n = 243, 5.3%), but frequency of GH/PE/eclampsia/ HELLP/AFLP was highest (67.1%), suggesting MgSO_4_ alone was mainly used eclampsia prevention during pregnancy (Supplementary Table S1). On the contrary, frequencies of PL/shortened CL/CI were significantly higher in the ritodrine alone group (79.6%) and the combined MgSO_4_ and ritodrine group (85.0%) compared to the MgSO_4_ alone group (35.8%) and the control group using neither MgSO_4_ nor ritodrine (30.4%). This suggests ritodrine alone or both ritodrine and MgSO_4_ in combination was mainly selected for PL therapy. Frequencies of hypoglycemia were significantly higher in women using ritodrine alone (41.4%) and using both MgSO_4_ and ritodrine (42.9%) than in controls (22.2%). Frequency of hyperkalemia was significantly higher in women using both MgSO_4_ and ritodrine (10.9%) than in controls (6.5%).

### Duration- and dose-dependent effects of ritodrine on hypoglycemia occurrence

Next, we evaluated the association of the duration, maximum rate of administration, final rate of administration just before cessation, and time from cessation to delivery for ritodrine with the occurrence of hypoglycemia using the “Hypoglycemia: Ritodrine-alone plus control set” (Supplementary Table S2). The risk of hypoglycemia was associated with long-term tocolysis (total administration periods ≥2 days [48 hours]). However, short-term tocolysis (<2 days [48 hours]) was not a risk factor for hypoglycemia. The maximum rate of injection or final rate of injection just before cessation of ritodrine did not show dose-dependence. The risk of hypoglycemia was related to cessation just before delivery; if ritodrine was stopped ≥4 hours before delivery, the aOR of hypoglycemia was almost two thirds of when stopped <4 hours before delivery.

### Duration- and dose-dependent effects of ritodrine or MgSO_4_ on hyperkalemia occurrence

Incidence rate of hyperkalemia in women with ritodrine alone was not different from that in women with MgSO_4_ alone. We then evaluated the association of tocolytic agents’ duration, maximum rate of administration, final rate of administration just before cessation, and time from cessation to delivery with hyperkalemia occurrence, using the “Hyperkalemia: Both ritodrine and MgSO_4_ plus control set” (Supplementary Table S3, S4). Hyperkalemia risk was associated with long-term tocolysis with ritodrine. In women in whom the maximum rate of injection or final rate of injection just before cessation of ritodrine was ≥170 μg/min, the risk was significantly higher than in those with no usage of ritodrine. Risk of hyperkalemia was related to the cessation of ritodrine just before delivery; if stopped ≥4 hours before delivery, hyperkalemia risk almost equaled no usage. As for MgSO_4_, there was no relationship between duration of administration, maximum rate of injection, or final rate of injection before cessation. However, risk of hyperkalemia was related to MgSO_4_ cessation just before delivery; hyperkalemia risk in women in whom MgSO_4_ was stopped ≥4 hours before delivery was not significantly different from that in women with no usage of ritodrine.

### Maternal and infantile characteristics based on presence/absence of cerebral palsy occurring 3 years after birth

Cerebral palsy occurred in 23 cases (0.5%) (Supplementary Table S5). Frequencies of placental abruption, delivery at <35 weeks of gestation, and Apgar score at 1 minute <3 were significantly higher in infants with cerebral palsy. Gestational weeks at delivery was earlier, and birth weight was also smaller. Due to this small sample size, we did not perform multivariable analysis.

### Maternal and infantile characteristics based on presence/absence of any neurological impairments occurring 3 years after birth

Neurological impairments occurred in 193 cases (4.5%) (Supplementary Table S6). Cerebral palsy was only 12%. Other impairments included developmental language disorder alone, low score of developmental quotient, autism spectrum disorder (autism), attention-deficit/hyperactivity disorder (ADHD), auditory disorder, visual impairment, epilepsy, or developmental coordination disorder. Frequencies of nulliparous women, GH/PE/eclampsia/ HELLP/AFLP, placenta abruption, delivery at <35 weeks of gestation, male infants, SGA infants, Apgar score at 1 minute <3, and hypoglycemia were higher in infants with neurological impairments. In addition, frequencies of PL/shortened CL/CI, or cervical incompetency, and twins/triplets were significantly lower in infants with neurological impairments. Median gestational weeks at delivery was earlier and birthweight was also smaller.

### Effects of various risk factors on occurrence of any neurological impairments

Neurological impairments were evaluated in 4,279 infants (Supplementary Table S7). Excluding 989 patients with missing data for 16 variables, a total of 3,290 patients underwent multivariable analysis to assess effects on occurrence of any neurological impairments with the following 16 risk factors: combination of MgSO_4_ and ritodrine, obstetrical complications, cesarean section, delivery at <35 wk, twins/triplets, infantile sex, SGA infants, large-for-gestational-age infants, Apgar score at 1 min <3, hypoglycemia at <48 h after birth, and hyperkalemia at <48 h after birth. Placental abruption, delivery at <35 weeks of gestation, male sex, SGA infants, and hypoglycemia were independent risk factors for the occurrence of any neurological impairments.

## Discussion

Our current large cohort study yielded three novel findings. First, neonatal hyperkalemia within 48 hours after birth was associated with the concomitant usage of ritodrine and MgSO_4_ among late preterm infants born at 32–36 gestational weeks. Second, neonatal hypoglycemia within 48 hours was associated with the usage of ritodrine alone or the concomitant usage of ritodrine and MgSO_4_; incidence of hypoglycemia was markedly higher in infants born to mothers with cessation of ritodrine just before delivery; in addition, incidence of hypoglycemia was higher in infants born to mothers with long-term tocolysis with ritodrine. Third, in infants born at 32–36 weeks of gestation, placental abruption, delivery at <35 weeks of gestation, male sex, SGA infants, and hypoglycemia within 48 hours after birth were independent risk factors for the occurrence of any neurological impairments.

In the current study, for the first time we have revealed concomitant usage of ritodrine and MgSO_4_ is associated with incidence of neonatal hyperkalemia within 48 hours after birth in neonates born at 32–36 gestational weeks, although we could not show an association between neonatal hyperkalemia and cerebral palsy. Suzuki^2^ analyzed data available in the Cause Analysis Report and demonstrated the relationship between delivery and cerebral palsy was unknown in 6.2% of nearly 800 cases of cerebral palsy. These infants showed marked characteristics: although considered normal, thereafter their condition changed suddenly and finally they developed severe cerebral palsy. Of these, 6 were cases of hypoglycemia and 3 of hyperkalemia, and in 2 cases of hyperkalemia with later occurrence of cerebral palsy, ritodrine and MgSO_4_ were concomitantly used. In addition, in 11 of 14 cases of unexpected neonatal hyperkalemia soon after birth, ritodrine and MgSO_4_ were also concomitantly used^3^. Our results further support the suggested association between concomitant usage of ritodrine and MgSO_4_ and neonatal hyperkalemia. It is well known neonatal hyperkalemia can cause electrocardiographic abnormalities including ventricular tachycardia^31^. Therefore, our results serve as a warning about concomitant usage of ritodrine and MgSO_4_ to prevent neonatal hyperkalemia. However, it is unknown why concomitant usage of ritodrine and MgSO_4_, but not ritodrine alone or MgSO_4_ alone, is associated with neonatal hyperkalemia. Hypermagnesemia might affect Na + /K + -ATPase^15,32^; however, to the best of our knowledge, there have been no cohort studies suggesting a relationship between ritodrine administration and neonatal hyperkalemia. Therefore, our clinical observations may suggest presence of synergy between ritodrine and hypermagnesemia to modify the Na + /K + -ATPase function.

Although we could not reveal the association between neonatal hypoglycemia and the later occurrence of cerebral palsy in this study, it is well-known that neonatal hypoglycemia increases the incidence of cerebral palsy^33,34^. Risk factors for cerebral palsy in infants with hypoglycemia are as follows: blood glucose levels <15 mg/dL, long duration of hypoglycemia, non-reassuring fetal status (NRFS), low Apgar score <5 at 1 min, neonatal seizure, pathological jaundice, and hypertensive disorders of pregnancy for the mother^35^. Ritodrine is well-known to induce hyperglycemia in mothers which can cause hypoglycemia in neonates^36,37^. In the current study, incidence of hypoglycemia was markedly higher in infants born to mothers with either long-term tocolysis of ritodrine or cessation just before delivery. Therefore, ritodrine might be one of the risk factors for cerebral palsy. Hyperkalemia was not associated with the occurrence of cerebral palsy. In addition, we found placental abruption, delivery at <35 weeks of gestation, and Apgar score at 1 minute <3 were associated with later occurrence of cerebral palsy. Placental abruption and early delivery are well-known risk factors for cerebral palsy^38^. Although the contribution of asphyxia to the overall incidence of cerebral palsy is relatively small^39^, metabolic acidosis in fetal umbilical cord arterial blood obtained at delivery with both pH <7.00 and base deficit ≥12 mmol/L would have been sufficient to cause cerebral palsy^40^.

In infants born at 32–36 weeks of gestation, placental abruption, delivery at <35 weeks of gestation, male sex, SGA infants, and hypoglycemia were independent risk factors for occurrence of any neurological impairments. Hyperkalemia was not an independent risk factor. Male sex is at higher risk of autism^41,42^, and may be at high risk of ADHD^43^; however, the association between male sex and cerebral palsy is controversal^44^. An SGA infant is a risk factor for cerebral palsy in moderate to late preterm infants^45^, and may be also associated with autism or ADHD^46,47^. Although our outcome of any neurological impairment was a composite outcome, and our data could be significantly biased due to a retrospective study design, our data support associations of male sex or an SGA infant on occurrence of neurological impairments including cerebral palsy, autism, or ADHD.

Our results warn about the concomitant usage of ritodrine and MgSO_4_ to prevent neonatal hyperkalemia, and also warn about long-term usage of ritodrine and its cessation just before delivery to prevent neonatal hypoglycemia. Furthermore, because these adverse events occurring outside NICUs are not well-recognized among obstetricians and neonatologists, this study could have a marked impact on modifying current neonatal management for infants born to mothers with tocolytic agents. Accordingly, neonatal assessment of hyperkalemia can be suggested in women with concomitant usage of ritodrine and MgSO_4_, and neonatal blood glucose monitoring can also be suggested in women with long-term tocolysis with ritodrine or cessation just before delivery.

The main strengths of our study include the following: (1) it is the first large cohort study evaluating associations between long-term tocolysis with both ritodrine and MgSO_4_, and neonatal hypoglycemia and hyperkalemia; (2) it presents the first evidence of a synergic effect between ritodrine and MgSO_4_ on occurrence of neonatal hyperkalemia; and (3) it is the first large cohort study investigating association of neonatal hyperkalemia and later occurrence of any neurodevelopmental impairments including cerebral palsy.

Our study has several limitations. First, it may be difficult to generalize results from this study with other countries. However, ritodrine hydrochloride and MgSO_4_ have a long usage history in prenatal care, and MgSO_4_ use is still widespread. The novel adverse events (neonatal hyperkalemia) due to concomitant use of ritodrine and magnesium sulfate should be added in the package insert of both MgSO_4_ and ritodrine hydrochloride. Second, this was a retrospective cohort study possibly resulting in high risk of selection bias. Although we targeted infants born at 32–36 gestational weeks, 19% of infants did not have data for either hypoglycemia or hyperkalemia possibly leading to bias. In addition, we could not confirm presence/absence of neurodevelopmental impairments in almost 7% of infants, resulting in underestimation of the associations between hypoglycemia/hyperkalemia and subsequent occurrence of neurodevelopmental impairments. However, in this large retrospective cohort study, we attempted to decrease systematic bias through secondary investigation for ritodrine and magnesium sulfate usage, and attempted to adjust possible confounding factors using multivariable logistic regression analysis. In addition, before using the nationwide database, we checked for inappropriate values and transformed them to missing values. The main outcomes of hypoglycemia and hyperkalemia were newly investigated in the secondary inquiry, and diagnoses of hypoglycemia and hyperkalemia were validated by investigating levels of glucose and potassium within 48 hours after birth. Therefore, although this study is a retrospective study using a large database, we believe reducing selection bias risk is feasible as it adjusting for possible confounding factors in the relationship between ritodrine/magnesium sulfate usage and neonatal hypoglycemia/hyperkalemia occurrence. Third, we could not collect data on hypoglycemia/hyperkalemia from 277 hospitals (78%). However, the incidences of GH/PE/eclampsia/HELLP/AFLP, placenta previa/low-lying placenta, DM, GDM, and cesarean section in infants in the current study were almost the same as those in infants not involved in the current study (Supplementary Table S8), indicating current subjects may have been appropriately extracted from a nationwide obstetrical database. Fourth, inclusion criteria for pregnant women with only late preterm infants may limit generalizability of our results. However, since long-term tocolysis with ritodrine and MgSO_4_ is often performed at 32–36 gestational weeks in Japan, and since tocolysis is not performed at ≥37 gestational weeks, we believe targeting at 32–36 gestational weeks may be appropriate for analyzing the relationship between tocolytic agents and occurrence of neonatal hypoglycemia and hyperkalemia.

## Methods

### Study design and participants

This was a retrospective cohort study of neonates born at 32–36 gestational weeks using a nationwide obstetrical database from 2014^48^. Because we needed to collect information on the infantile prognosis until almost 3 years old, it was followed by a secondary survey conducted in Japan in 2017–2018. In previous case series of hyperkalemia neonates, 50% (7/14) were born at 32–36 gestational weeks^3–14^. Neonates are usually managed on obstetrical wards in Japan if they have either ≥2,000 g birthweight or are at ≥35 gestational weeks at delivery. Therefore, we speculated such neonates might have developed cerebral palsy due to possible delay of the detection of hyperkalemia, because in neonates on obstetrical wards electrolyte abnormalities are not checked routinely unless they show symptoms. In addition, hyperkalemia often occurs in neonates born at <32 gestational weeks; therefore, exclusion of such early preterm infants may facilitate analysis of the association between tocolytic agents and hyperkalemia occurrence. Thus, we decided to investigate neonates born in a relatively late preterm period (32–36 gestational weeks), to evaluate the possible relationship between tocolytic agents and neonatal hyperkalemia. For hypoglycemia, Suzuki^2^ analyzed data available in the Cause Analysis Report, and determined 5 of 6 neonates (83%) who had hypoglycemia suspected associated with development of cerebral palsy were born at ≥37 gestational weeks. However, we did not include neonates born at ≥37 gestational weeks in our analysis since assessment of hypoglycemia was not part of our routine examinations.

We received approval from the JSOG Clinical Research Ethics Committee to use a nationwide obstetrical database from 2014 (No. JSOG2017–51), and also received approval from the JSPNM Clinical Research Ethics Committee for execution of the current study in the survey group to study the effects of tocolytic agents on neonatal adverse events (No. JSPNM2017–1). Then, we requested the directors of Departments of Obstetrics and Gynecology in 355 hospitals that had registered in the nationwide obstetrical database from 2014 (total stillbirths and infants: n = 220,052; those born at 32–36 gestational weeks: n = 24,943) to cooperate with the current study. Finally, 78 directors consented to this study, and kindly secured the cooperation of neonatologists in each hospital. A research investigator and research team members in each hospital gained approval for this study from each Clinical Research Ethics Review Committee. All methods in this retrospective study were performed in accordance with the relevant guidelines (Ethical Guidelines for Medical and Health Research Involving Human Subjects). Because this study is a retrospective study, it was very difficult to gain appropriate informed consents from each subject. Therefore, we gained consents using opt-out, which is a way for investigators to give subjects an opportunity to refuse to participate in this study by announcing the detail of this study in each participating institute. The Survey Committee constructed input pages for survey data on the Web system. Data were collected within 1 year after the approval of the current study.

**Figure 1:**
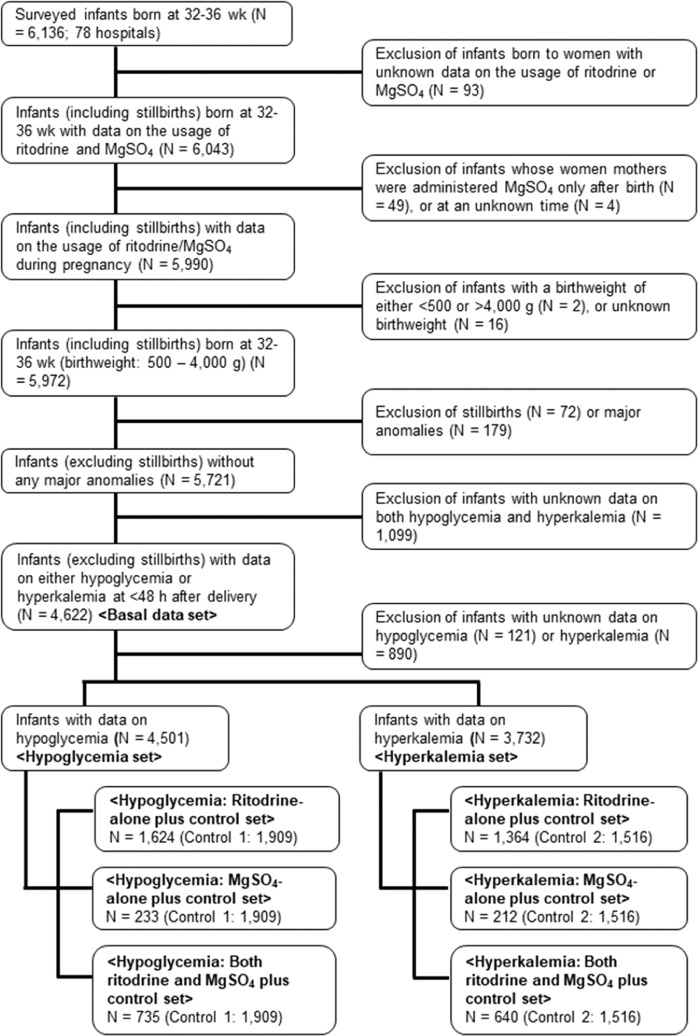
Patient Flowchart. “Basal data set” (N = 4,622) was created from 6,136 surveyed infants born at 32–36 gestational weeks. “Hypoglysemia set” (N = 4,501) was created from “Basal data set” after excluding 121 infants with unknown data on hypoglycemia; and “Hyperkalemia set” (N = 3,732) was created from “Basal data set” after excluding 890 infants with unknown data on hyperkalemia. “Hypoglycemia set” was divided into “Hypoglycemia: Ritodrine-alone plus control set” (Ritodrine-alone [N = 1,624] and control 1 without either ritodrine or MgSO_4_ [N = 1,909]), “Hypoglycemia: MgSO_4_-alone plus control set” (MgSO_4_-alone [N = 233] and control 1), and “Hypoglycemia: Both ritodrine and MgSO_4_ plus control set” (Both ritodrine and MgSO_4_ [N = 735] and control 1). “Hyperkalemia set” was also divided into “Hyperkalemia: Ritodrine-alone plus control set” (Ritodrine-alone [N = 1,364] and control 2 without either ritodrine or MgSO_4_ [N = 1,516]), “Hyperkalemia: MgSO_4_-alone plus control set”, (MgSO_4_-alone [N = 212] and control 2), and “Hyperkalemia: Both ritodrine and MgSO_4_ plus control set” (Both ritodrine and MgSO_4_ [N = 640] and control 2).

We excluded the following infants from our survey: (1) born to women with unknown data on the usage of ritodrine or MgSO_4_, (2) whose mothers were administered MgSO_4_ only after birth or at an unknown time, (3) with a birthweight of either <500 or>4,000 g, or unknown birth weight, (4) with stillbirth or major anomalies (chromosomal anomalies, neonatal abnormalities, conditions probably contributing to impaired neurodevelopment, conditions requiring emergency surgery soon after delivery, and lethal conditions), and (5) with unknown data on both hypoglycemia and hyperkalemia (Fig. 1). The remaining 4,622 infants with data on either hypoglycemia or hyperkalemia at <48 hours after delivery were analyzed.

### Collection of new variables by the secondary survey

The Survey Committee used a nationwide obstetrical database from 2014, which included 314 variables on maternal and neonatal information^48^. The input data in all variables were initially automatically checked using internalized data check scripts, and data input staff were informed of possible incorrect data. However, since the database was built using data from 355 institutes, there were inappropriate data in the database. Then, one author (A.O.) attempted to validate the database. The initial number of cases in the database was 24,960, but we found that 17 cases were duplicated; after exclusion the remaining 24,943 cases were used. Next, we determined inappropriate values for maternal height, pre-pregnancy maternal body weight, maternal body weight at delivery, maternal age, bleeding amounts, gestational weeks at premature rupture of the membranes, disseminated intravascular coagulation (DIC) score, neonatal birth weight, neonatal birth height, neonatal head circumference, Apgar score at 1 minute (min), Apgar score at 5 min, pH of umbilical artery, placental weight, and umbilical cord length. We finally transformed the inappropriate values to missing values.

The Survey Committee extracted the following 6 variables: facility name, anonymization number, date of birth, gestational weeks and days at delivery, birth weight, and neonatal sex. The committee members collaboratively decided survey items for the secondary survey. In the survey for ritodrine they were: presence/absence of injections, medical product name, total administration days (6 codes), maximum infusion speed (8 codes), final infusion speed (8 codes), and interval (hours) from cessation of ritodrine to delivery (6 codes). In the MgSO_4_ survey they were: presence/absence of injections, medical product name, total administration days (6 codes), maximum infusion speed (8 codes), final infusion speed (8 codes), interval hours from cessation of MgSO_4_ to delivery (6 codes), and administration period (pre-delivery alone, post-delivery alone, both pre- and post-delivery, unknown). In the survey for neonatologists they were: presence/absence of admission to NICU, causes for admission to NICU, presence/absence of measurements of magnesium concentrations in umbilical cord, concentration of magnesium in umbilical cord, presence/absence of measurements of blood sugar within 48 hours after delivery, blood sugar level at the nadir (mg/dL), presence/absence of hypoglycemia defined as <40 mg/dL^49^, timing of the nadir blood sugar level (5 codes), presence/absence of measurements of potassium concentrations within 48 hours after delivery, potassium level at the maximum (mEq/L), presence/absence of hyperkalemia defined as >6.5 mEq/L^50^, timing of the maximum potassium level (5 codes), three consecutive potassium levels just after the occurrence of hyperkalemia, infantile prognosis at almost 3 years (4 codes: death, presence of abnormal neurological findings, absence of abnormal neurological findings, unknown), detailed information on the disease or condition leading to abnormal neurological findings, and date of judgment of infantile prognosis.

### Primary/secondary outcomes and risk factors

Primary outcomes were the occurrence of hyperkalemia and hypoglycemia. Secondary outcomes were cerebral palsy, and any neurodevelopmental impairments including cerebral palsy occurring 3 years after birth. Cerebral palsy was judged by the senior pediatrician (S. K.) who was involved neither in data acquisition nor in database construction. Cerebral palsy was defined as a non-progressive, non-transient central nervous system disorder characterized by abnormal muscle tone in at least 1 extremity and abnormal control of movement and posture^51^.

Based on both clinical relevance and univariate analysis, risk factors for occurrence of hypoglycemia or hyperkalemia were: obstetrical complications, cesarean section, MgSO_4_ usage during pregnancy, ritodrine usage, gestational weeks at delivery, birth weight, multiple pregnancy, infantile sex, SGA and large-for-gestational-age defined as an infant with weight below the 10th percentile or ≥ the 90th percentile of gestational age^52^, and Apgar score at 1 min <3. The above-mentioned 14 risk factors plus hypoglycemia and hyperkalemia were also determined risk factors for cerebral palsy or other neurological impairments.

### Statistical analysis

Continuous variables are shown as the median (interquartile range) because of non-normal distributions of gestational weeks and birth weight at 32–36 gestational weeks, and binary and categorical variables are shown as n (%). The associations of ritodrine/MgSO_4_ usage during pregnancy and the occurrence of infantile hypoglycemia/hyperkalemia within 48 hours after delivery were analyzed using Fisher’s exact test or the χ^2^ test, followed by univariate logistic regression analyses. Then, multivariable regression analyses were performed while adjusting for confounding variables. Because the primary outcomes of hyperkalemia and hypoglycemia occurred in >200 cases, we judged we could use a maximum of 20 risk and/or confounding factors in the multivariable models. All analyses were performed using IBM SPSS Statistics (version 25 for Windows) and R (EZR ver. 1.37)^53^. Level of *p* < 0.05 was considered significant.

## Supplementary information

Supplementary Information.

## Data Availability

Data and materials used in this study are available upon reasonable request to the corresponding author and under a collaboration agreement.
