# Supplementary material for: Synergic interaction between ritodrine and magnesium sulfate on the occurrence of critical neonatal hyperkalemia: A Japanese nationwide retrospective cohort study
Source: Sci Rep. 2020 May 8;10:7804. doi: 10.1038/s41598-020-64687-w (PMC7210882; doi:10.1038/s41598-020-64687-w)
Supplement: Supplementary file 1 — Supplementary Information. [file 41598_2020_64687_MOESM1_ESM.pdf]

Synergic interaction between ritodrine and magnesium sulfate on the occurrence of critical neonatal hyperkalemia: A Japanese nationwide retrospective cohort study

Yukari Yada[1], Akihide Ohkuchi[2], Katsufumi Otsuki[3], Keiji Goishi[4], Mari Takahashi[5], Naohiro Yonemoto[6], Shigeru Saito[7], Satoshi Kusuda[8], on behalf of "The Survey Group Studying the Effects of Tocolytic Agents on Neonatal Adverse Events in Japan Society of Perinatal and Neonatal Medicine"[5]

[1]Department of Pediatrics, Jichi Medical University School of Medicine, Tochigi, Japan; [2]Department of Obstetrics and Gynecology, Jichi Medical University School of Medicine, Tochigi, Japan; [3]Department of Obtetrics and Gynecology, Showa University Koto Toyosu Hospital, Tokyo, Japan; [4]Department of Pediatrics, National Center for Global Health and Medicine, Tokyo, Japan; [5]Japan Society of Perinatal and Neonatal Medicine, Tokyo, Japan; [6]Department of Psychopharmacology, National Center of Neurology and Psychiatry, Tokyo, Japan; [7]Department of Obstetrics and Gynecology, University of Toyama, Toyama, Japan; [8]Department of Pediatrics, Kyorin University, Tokyo, Japan.

Corresponding author:

Akihide Ohkuchi, MD

Department of Obstetrics and Gynecology, Jichi Medical University School of Medicine, 3311-1 Yakushiji, Shimotsuke-shi, Tochigi 329-0498, Japan, [okuchi@jichi.ac.jp], 81-285-58-7376.

Consortia representative:

Satoshi Kusuda[5], MD

Japan Society of Perinatal and Neonatal Medicine, 2-30 Ichigayahonmuracho, Shinjuku-ku, Tokyo 162-0845 Japan, [skusudag@gmail.com], 81-3-5228-2074.

Consortia

Authors' list for the Survey Group Studying the Effects of Tocolytic Agents on Neonatal Adverse Events in Japan Society of Perinatal and Neonatal Medicine

Hajime Ota[9], Kiyotaka Kosugiyama[9], Kazuhiko Okuyama[10], Masato Mizushima[10], Hideaki Negishi[11], Shinichi Koshida[11], Mayumi Kasai[12], Motonari Okabe[13], Akira Sato[13], Hiroyuki Adachi[13], Michio Banzai[14], Kazuhiro Akaba[14], Rika Suzuki[15], Naohisa Ishibashi[15], Takashi Watanabe[16], Yoshio Kasuga[17], Takashi Kameda[18], Toru Fujiu[18], Takeshi Takagi[19], Kenichi Maruyama[19], Masahiko Higashino[20], Tomomi Naito[20], Yoshimasa Kamei[21], Tetsuya Kunikata[21], Yoshinori Iitsuka[22], Harumi Otsuka[22], Yuka Yamamoto[23], Mie Yamada[24], Masaki Daigo[24], Hironobu Hyodo[25], Ayumi Sato[26], Noriko Kataoka[27], Satoko Yamanaka[27], Aya Okahashi[28], Yuki Kojima[29], Shigenori Kabashima[29], Yoshie Nakamura[29], Rina Okuno[29], Seiko Hirose[29], Koichi Sugahara[30], Satsuki Okamoto[30], Sumiko Hara[31], Wakako Shima[31], Takeshi Suzuki[32], Hideyuki Kagawa[33], Kenichiro Fujioka[33], Akiko Kurasaki[34], Ayako Miura[34], Isamu Hokuto[34], Toru Arase[35], Nobuhiko Taguchi[35], Kazuki Sekiguchi[36], Tomoyo Matsuo[37], Emi Ohnuma[37], Kana Fujiwara[37], Miyuki Ogawa[38], Azusa Uozumi[38], Noriyuki Yokomichi[39], Akane Hirose[39], Mika Okuda[40], Ayako Fukuyama[40], Hitoshi Ishimoto[41], Kanako Mitsuzuka[41], Shinya

Kondo[42], Miyuki Kitazawa[43], Norihiko Kikuchi[44], Yumiko Miyashita[45], Chiharu Tsutsumi[45], Shuhei Terada[46], Shigeru Ohki[46], Takakazu Kawamura[47], Masako Yasuda[48], Yoshiki Soeno[48], Takumi Kurabayashi[49], Yoshihisa Nagayama[49], Satoshi Yoneda[7], Tomomi Shiga[50], Seiji Hayashi[51], Hiroyuki Tsuda[52], Makoto Oshiro[52], Takafumi Ushida[53], Teruyuki Mizutani[53], Hideyuki Asada[53], Ryousuke Miura[53], Ryo Tanaka[53], Noriko Kato[54], Yuko Sasaki[54], Takehiko Yokoyama[54], Takako Hirooka[54], Takaharu Yamada[54], Kaori Maruwaka[54], Syunsuke Nagara[54], Satoko Fukaya[54], Mari Koroki[54], Taihei Tanaka[54], Shigehiko Morikawa[55], Shigeru Honda[55], Haruki Sassa[56], Takeshi Sahashi[56], Hiroko Torii[57], Tadahiro Yasuo[58], Nozomi Kuriyama[58], Juzo Okada[59], Moe Kano[59], Noriyoshi Oki[59], Mieko Inagaki[59], Yousuke Mizuno[59], Masayo Fujisaka[59], Akihiro Takatera[59], Takeo Mure[59], Katsuhiko Yoshii[59], Yasuko Furuichi[60], Akiko Kanto[61], On Fukui[62], Shusaku Hayashi[63], Hitomi Ono[63], Eri Fujikawa[63], Masayuki Someya[63], Makiko Ikeda[63], Kentaro Nakanishi[63], Akiko Yamashita[63], Haruna Kawaguchi[63], Ryo Yamamoto[63], Jun Sasahara[63], Takeshi Kanagawa[63], Satoshi Yamamoto[63], Yosuke Imanishi[63], Misuzu Yoshida[63], Eri Yano[63], Ayumi Murayama[63], Kazue Morikawa[63], Natsuko Tabata[63], Ryosuke Araki[63], Eriko Iwasaki[63], Narutaka Mochizuki[63], Akiko Kobayashi[63], Akiko Takeda[64], Akiko Kobayashi[64], Masaya Hirose[65], Nao Taguchi[65], Hiroshi Sato[65], Kenji Oida[65], Rie Sakai[65], Saeko Imai[65], Reona Shiro[65], Minami Okudate[65], Yoko Matsuda[65], Yoshinobu Nishida[65], Aya Toyofuku[66], Shigeto Hara[66], Hiroko Kurioka[67], Tomoya Mizunoe[68], Syouhei Eto[68], Takahiro Nobuzane[69], Kousyou Higuchi[69], Terumi Miwa[70], Keiko Hasegawa[70], Yuko Matsubara[71], Masaaki Ohta[71], Takafumi Watanabe[72], Takako Ohmaru-Nakanishi[73], Kana Kashinoura[74], Maki Goto[75], Hiroshi Kanda[75], Kiyomi Tsukimori[76], Yasushi Takahata[76], Makoto Nomiyama[77], Toshimitsu Takayanagi[77], Syuichiro Yoshimura[78], Kouhei

Kotera[78], Hisanobu Fukuda[78], Hiroko Hiraki[78], Noriko Nagata[78], Kazuhisa Nakashima[78], Junya Miyoshi[79], Takafumi Obara[79], Kentaro Kai[80], Yuichi Furukawa[80], Satoshi Eto[80], Tomoko Oishi[80], Misaki Nakashima[80], Aya Yamauchi[81], Yuki Kodama[82], Takako Ohata[83], Haruka Arakaki[83], Kei Miyakoshi[84], Mariko Hida[84]

[9]Teine Keijinkai Hospital, Hokkaido, Japan. [10]Sapporo City General Hospital, Hokkaido, Japan. [11]Japanese Red Cross Hospital Kitami, Hokkaido, Japan. [12]Iwate Prefectural Central Hospital, Iwate, Japan. [13]Akita University, Akita, Japan. [14]Yamagata Saisei Hospital, Yamagata, Japan. [15]Ohara General Hospital, Fukushima, Japan. [16]Haga Red Cross Hospital, Tochigi, Japan. [17]Japanese Red Cross Ashikaga Hospital, Tochigi, Japan. [18]Gunma University, Gunma, Japan. [19]Gunma Children's Medical Center, Gunma, Japan. [20]Saiseikai Kawaguchi General Hospital, Saitama, Japan. [21]Saitama Medical University Hospital, Saitama, Japan. [22]Chiba Kaihin Municipal Hospital, Chiba, Japan. [23]Juntendo University Urayasu Hospital, Chiba, Japan. [24]San-Ikukai Hospital, Tokyo, Japan. [25]Tokyo Metropolitan Bokutoh Hospital, Tokyo, Japan. [26]NTT Medical Center Tokyo, Tokyo, Japan. [27]International Catholic Hospital, Tokyo, Japan. [28]Nihon University Itabashi Hospital, Tokyo, Japan. [29]Tachikawa Sougo General Hospital, Tokyo, Japan. [30]Fussa Hospital, Tokyo, Japan. [31]Tokyo Adventist Hospital, Tokyo, Japan. [32]Kawasaki Municipal Hospital, Kanagawa, Japan. [33]Kanto Rosai Hospital, Kanagawa, Japan. [34]St. Mariannna university School of Medicine, Kanagawa, Japan. [35]Keiyu Hospital, Kanagawa, Japan. [36]Kitasato University, Kanagawa, Japan. [37]Saiseikai Yokohamashi Nanbu Hospital, Kanagawa, Japan. [38]Yokohama City University Hospital, Kanagawa, Japan. [39]St. Marianna University School of Medicine, Yokohama City Seibu Hospital, Kanagawa, Japan. [40]National Hospital Organization

Yokohama Medical Center, Kanagawa, Japan. [41]Tokai University, Kanagawa, Japan. [42]Kanagawa Children's Medical Center, Kanagawa, Japan. [43]Shinonoi General Hospital, Nagano, Japan. [44]Shinshu University, Nagano, Japan. [45]Shizuoka City Shimizu Hospital, Shizuoka, Japan. [46]Seirei Hamamatsu General Hospital, Shizuoka, Japan. [47]Shizuoka Children's Hospital, Shizuoka, Japan. [48]Nagaoka Red Cross Hospital, Niigata, Japan. [49]Niigata City General Hospital, Niigata, Japan. [50]Gifu University, Gifu, Japan. [51]Okazaki City Hospital, Aichi, Japan. [52]Japanese Red Cross Nagoya Daiichi Hospital, Aichi, Japan. [53]Nagoya University, Aichi, Japan. [54]Japanese Red Cross Nagoya Daini Hospital, Aichi, Japan. [55]Komaki City Hospital, Aichi, Japan. [56]Ichinomiya Municipal Hospital, Aichi, Japan. [57]Kusatsu General Hospital, Shiga, Japan. [58]Japanese Red Cross Kyoto Daiichi Hospital, Kyoto, Japan. [59]Chibune General Hospital, Osaka, Japan. [60]Higashiosaka City Medical Center, Osaka, Japan. [61]Kindai University, Osaka, Japan. [62]Sakai City Medical Center, Osaka, Japan. [63]Osaka Women's and Children's Hospital, Osaka, Japan. [64]National Hospital Organization Kobe Medical Center, Hyogo, Japan. [65]Hyogo Prefectural Amagasaki General Medical Center, Hyogo, Japan. [66]Japanese Red Cross Wakayama Medical Center, Wakayama, Japan. [67]Shimane Prefectural Central Hospital, Shimane, Japan. [68]National Hospital Organization Kure Medical Center, Hiroshima, Japan. [69]Chugoku Rosai Hospital, Hiroshima, Japan. [70]Yamaguchi Grand Medical Center, Yamaguchi, Japan. [71]Ehime University, Ehime, Japan. [72]Kochi Health Sciences Center, Kochi, Japan. [73]Hamanomachi Hospital, Fukuoka, Japan. [74]National Hospital Organization Kyushu Medical Center, Fukuoka, Japan. [75]Iizuka Hospital, Fukuoka, Japan. [76]Fukuoka Children's Hospital, Fukuoka, Japan. [77]National Hospital Organization Saga Hospital, Saga, Japan. [78]Nagasaki Harbor Medical Center, Nagasaki, Japan. [79]Japanese Red Cross Kumamoto Hospital, Kumamoto, Japan. [80]Nakatsu

Municipal Hospital, Oita, Japan. [81]Miyazaki Prefectural Nobeoka Hospital, Miyazaki, Japan. [82]University of Miyazaki, Miyazaki, Japan. [83]Okinawa Chubu Hospital, Okinawa, Japan. [84]Keio University Hospital, Tokyo, Japan.

Supplemantary Table S1 Maternal and infantile characteristics involving 4,622 infants born at 32-36 weeks of gestation, with the combination of $MgSO_4$ and ritodrine

Characteristics	Neither $MgSO_4$ nor ritodrine (Group 1: G1) (n = 1,951)	$MgSO_4$ alone (Group 2: G2) (n = 243)	Ritodrine alone (Group 3: G3) (n = 1,679)	Both $MgSO_4$ and ritodrine (Group 4: G4) (n = 749)	Missing data	*P* -value	Significant pair(s)[a]
Maternal characteristics							
Age (yr)	33.5 (29.5 - 37.5)	34.5 (30.5 - 37.5)	33.5 (29.5 - 36.5)	32.5 (28.5 - 35.5)	2	<0.001	All excluding G1 vs G2, G1 vs G3
Nulliparity	974 (49.9)	132 (54.3)	843 (50.2)	400 (53.4)	0	0.254	
Obstetrical complications							
PL/shortened CL/CI	594 (30.4)	87 (35.8)	1,334 (79.5)	637 (85.0)	0	<0.001	All excluding G1 vs G2
pPROM	550 (28.2)	26 (10.7)	502 (29.9)	149 (19.9)	0	<0.001	All excluding G1 vs G3
GH/PE/eclampsia/HELLP/AFLP	357 (18.3)	163 (67.1)	105 (6.3)	76 (10.1)	0	<0.001	All
Placental abruption	82 (4.2)	2 (0.8)	42 (2.5)	11 (1.5)	0	<0.001	G1 vs G3, G1 vs G4
Placenta previa/Low-lying placenta	92 (4.7)	5 (2.1)	135 (8.0)	70 (9.3)	0	<0.001	All excluding G1 vs G2 and G3 vs G4
DM	24 (1.2)	9 (3.7)	12 (0.7)	2 (0.3)	0	<0.001	G1 vs G2, G2 vs G3, G2 vs G4
GDM	97 (5.0)	39 (16.0)	63 (3.8)	47 (6.3)	0	<0.001	All excluding G1 vs G3, G1 vs G4
Possible maternal risk factors for either hypoglycemia or hyperkalemia							
Cesarean section	1,194/1,926 (62.0)	182/242 (75.2)	1,040/1,660 (62.7)	485/743 (65.3)	51	<0.001	G1 vs. G2, G2 vs. G3, G2 vs. G4
Possible children's risk factors for either hypoglycemia or hyperkalemia							
Gestational weeks at delivery	35.9 (34.8 - 36.5)	34.8 (33.6 - 36.1)	35.5 (34.2 - 36.4)	34.9 (33.6 - 36.1)	0	<0.001	All excluding G2 vs. G4
Delivery at <35 wk	579 (29.7)	137 (56.4)	665 (39.6)	392 (52.3)	0	<0.001	All excluding G2 vs. G4
Birthweight (g)	2,218 (1,915 - 2,500)	1,894 (1,628 - 2,230)	2,212 (1,940 - 2,462)	2,140 (1,827 - 2,400)	0	<0.001	All excluding G1 vs. G3
Twins/Triplets	452 (23.2)	53 (21.8)	546 (32.5)	314 (41.9)	0	<0.001	All excluding G1 vs. G2
Sex: male	1,071 (54.9)	127 (52.3)	951 (56.6)	432/748 (57.8)	1	0.319	
SGA infants	272 (13.9)	49 (20.2)	160 (9.5)	69 (9.2)	0	<0.001	All excluding G1 vs. G2 and G3 vs. G4
Large-for-gestational-age infants	30 (1.5)	2 (0.8)	14 (0.8)	7 (0.9)	0	0.203	
Apgar score at 1 min <3	65/1,946 (3.3)	5 (2.1)	39 (2.3)	29 (3.9)	5	0.105	
Primary outcomes							
Hypoglycemia at <48 h after birth	423/1,909 (22.2)	47/233 (20.2)	673/1,624 (41.4)	315/735 (42.9)	121	<0.001	All excluding G1 vs. G2 and G3 vs. G4
Hyperkalemia at <48 h after birth	98/1,516 (6.5)	12/212 (5.7)	104/1,364 (7.6)	70/640 (10.9)	890	0.003	G1 vs. G4

Abbreviations: yr, years old; PL, preterm labor; CL, cervical length; CI, cervical incompetency; GH, gestational hypertension; PE, preeclampsia; HELLP, hemolysis, elevated liver enzymes, and low platelets; AFLP, acute fatty liver of pregnancy; DM, diabetes mellitus; GDM, gestational diabetes mellitus; SGA, small-for-gestational-age; min, minutes; h, hour(s).

Continuous variables are shown as median (interquartile range), and discrete variables are shown as n (%).

This analysis was performed using the "Basal data set".

a: Multiple group comparisons were performed using the χ^2 test followed by Bonferroni test, or the Kruskal-Wallis test followed by Bonferroni test.

Supplementary Table S2 Association of duration, maximum rate of administration, final rate of administration just before cessation, and time from cessation to delivery for ritodrine with hypoglycemia occurrence in 1,624 women with ritodrine alone and 1,909 women without tocolysis

Risk factors	Incidence of hypoglycemia		Univariable analysis			Multivariable analysis[a]		
			Crude odds ratio (95% CI)		*P*-value	Adjusted odds ratio (95% CI)		*P*-value
For ritodrine hydrochloride alone								
Duration of administration	(Missing values: 11)							
No usage	423/1,909	(22.2%)	1			1		
<48 h	74/319	(23.2%)	1.06	(0.80 - 1.41)	0.680	1.18	(0.88 - 1.59)	0.275
**2 to 6 days**	85/261	(32.6%)	1.70	(1.28 - 2.25)	<0.001	**1.94**	**(1.44 - 2.61)**	<0.001
**7 to 13 days**	122/254	(48.0%)	3.25	(2.48 - 4.25)	<0.001	**3.46**	**(2.61 - 4.59)**	<0.001
**14 to 27 days**	162/330	(49.1%)	3.39	(2.66 - 4.31)	<0.001	**3.36**	**(2.61 - 4.33)**	<0.001
**≥28 days**	229/449	(51.0%)	3.66	(2.95 - 4.53)	<0.001	**3.52**	**(2.79 - 4.43)**	<0.001
Maximum rate of injection (μg/min)	(Missing values: 49)							
No usage	423/1,909	(22.2%)	1			1		
**-79**	250/626	(39.9%)	2.34	(1.93 - 2.83)	<0.001	**2.34**	**(1.91 - 2.88)**	<0.001
**80 - 169**	275/659	(41.7%)	2.52	(2.08 - 3.04)	<0.001	**2.64**	**(2.16 - 3.24)**	<0.001
**170 -**	123/290	(42.4%)	2.59	(2.00 - 3.35)	<0.001	**2.85**	**(2.17 - 3.74)**	<0.001
Final rate of injection (μg/min) just before cessation	(Missing values: 32)							
No usage	423/1,909	(22.2%)	1			1		
**-79**	320/783	(40.9%)	2.43	(2.03 - 2.90)	<0.001	**2.48**	**(2.05 - 3.00)**	<0.001
**80 - 169**	237/566	(41.9%)	2.53	(2.07 - 3.09)	<0.001	**2.68**	**(2.17 - 3.31)**	<0.001
**170 -**	105/243	(43.2%)	2.67	(2.03 - 3.52)	<0.001	**2.88**	**(2.16 - 3.85)**	<0.001
Time (h) from cessation to delivery	(Missing values: 38)							
No usage	423/1,909	(22.2%)	1			1		
**0 - 1.9**	395/823	(48.0%)	3.24	(2.72 - 3.86)	<0.001	**2.95**	**(2.44 - 3.56)**	<0.001
**2.0 - 3.9**	71/149	(47.7%)	3.20	(2.28 - 4.49)	<0.001	**3.39**	**(2.38 - 4.83)**	<0.001
**≥4.0**	191/614	(31.1%)	1.59	(1.30 - 1.94)	<0.001	**1.99**	**(1.59 - 2.48)**	<0.001

Abbreviations: CI, confidence interval; h, hour(s); min, minute.

This analysis was performed using the "Hypoglycemia: Ritodrine-alone plus control set" derived from "Hypoglycemia set".

a: Multivariable analyses were adjusted by the following 13 variables: obstetrical complications (pPROM, GH/PE/eclampsia/HELLP/AFLP, placental abruption, placenta previa/low-lying placenta, DM, and GDM), cesarean section, delivery at <35 wk, twins/triplets, infantile sex, SGA infants,large-for-gestational-age infants, and Apgar score at 1 min <3. For "duration of administration", "maximum rate of injection", "final rate of injection just before cessation", and "time from cessation to delivery", excluding 60, 98, 81, and 86 patients with missing data for 13 variables, totals of 3,473, 3,435, 3,452, and 3,447 patients were used for multivariable analysis, respectively. Abbreviations: pPROM, preterm premature rupture of the membranes; GH, gestational hypertension; PE, preeclampsia; HELLP, hemolysis, elevated liver enzymes, and low platelets; AFLP, acute fatty liver of pregnancy; DM, diabetes mellitus; GDM, gestational diabetes mellitus; wk, gestational weeks; SGA, small-for-gestational-age.

Supplementary Table S3 Association of duration, maximum rate of administration, final rate of administration just before cessation, and time from cessation to delivery for ritodrine with hyperkalemia occurrence in 640 women with both ritodrine and $MgSO_4$ and 1,516 women without tocolysis

Risk factors	Incidence of hypoglycemia		Univariable analysis			Multivariable analysis[a]		
			Crude odds ratio (95% CI)		*P*-value	Adjusted odds ratio (95% CI)		*P*-value
For ritodrine (with $MgSO_4$)								
Duration of administration	(Missing values: 9)							
No usage	98/1,516	(6.5%)	1			1		
<48 h	5/51	(9.8%)	1.57	(0.61 - 4.05)	0.348	1.35	(0.52 - 3.54)	0.542
2 to 6 days	6/78	(7.7%)	1.21	(0.51 - 2.84)	0.669	1.06	(0.44 - 2.55)	0.892
**7 to 13 days**	12/83	(14.5%)	2.45	(1.28 - 4.66)	0.007	**2.12**	**(1.08 - 4.15)**	**0.029**
14 to 27 days	9/93	(9.7%)	1.55	(0.76 - 3.18)	0.231	1.31	(0.63 - 2.73)	0.474
**≥28 days**	37/326	(11.3%)	1.85	(1.24 - 2.76)	0.002	**1.64**	**(1.06 - 2.55)**	**0.026**
Maximum rate of injection (μg/min)	(Missing values: 11)							
No usage	98/1,516	(6.5%)	1			1		
-79	9/97	(9.3%)	1.48	(0.72 - 3.03)	0.283	1.19	(0.57 - 2.47)	0.650
80 - 169	15/184	(8.2%)	1.28	(0.73 - 2.26)	0.387	1.16	(0.65 - 2.07)	0.627
**170 -**	44/348	(12.6%)	2.09	(1.44 - 3.05)	<0.001	**1.82**	**(1.21 - 2.75)**	**0.004**
Final rate of injection (μg/min) just before cessation	(Missing values: 13)							
No usage	98/1,516	(6.5%)	1			1		
-79	16/161	(9.9%)	1.60	(0.92 - 2.78)	0.099	1.4	(0.79 - 2.48)	0.243
80 - 169	14/183	(7.7%)	1.20	(0.67 - 2.15)	0.542	0.99	(0.53 - 1.84)	0.973
**170 -**	37/283	(13.1%)	2.18	(1.46 - 3.25)	<0.001	**1.93**	**(1.25 - 2.99)**	**0.003**
Time (h) from cessation to delivery	(Missing values: 21)							
No usage	98/1,516	(6.5%)	1			1		
**0 - 1.9**	41/338	(12.1%)	2.00	(1.36 - 2.94)	<0.001	**1.77**	**(1.17 - 2.70)**	**0.007**
2.0 - 3.9	9/63	(14.3%)	2.41	(1.16 - 5.03)	0.019	1.80	(0.80 - 4.03)	0.154
≥4.0	17/218	(7.8%)	1.22	(0.72 - 2.09)	0.460	1.11	(0.63 - 1.95)	0.721

Abbreviations: $MgSO_4$, magnesium sulfate; CI, confidence interval; h, hour(s); min, minute.

This analysis was performed using the "Hyperkalemia: Both ritodrine and $MgSO_4$ plus control set" derived from "Hyperkalemia set".

a: Multivariable analyses were adjusted by the following 13 variables: obstetrical complications (pPROM, GH/PE/eclampsia/HELLP/AFLP, placental abruption, placenta previa/low-lying placenta, DM, and GDM), cesarean section, delivery at <35 wk, twins/triplets, infantile sex, SGA infants,large-for-gestational-age infants, and Apgar score at 1 min <3. For "duration of administration", "maximum rate of injection", "final rate of injection just before cessation", and "time from cessation to delivery", excluding 36, 38, 40, and 48 patients with missing data for 13 variables, totals of 2,120, 2,118, 2,116, and 2,108 patients were used for multivariable analysis, respectively. Abbreviations: pPROM, preterm premature rupture of the membranes; GH, gestational hypertension; PE, preeclampsia; HELLP, hemolysis, elevated liver enzymes, and low platelets; AFLP, acute fatty liver of pregnancy; DM, diabetes mellitus; GDM, gestational diabetes mellitus; wk, gestational weeks; SGA, small-for-gestational-age.

Supplementary Table S4 Association of duration, maximum rate of administration, final rate of administration just before cessation, and time from cessation to delivery for $MgSO_4$ with hyperkalemia occurrence in 640 women with both ritodrine and $MgSO_4$ and 1,516 women without tocolysis

Risk factors	Incidence of hypoglycemia		Univariable analysis			Multivariable analysis[a]		
			Crude odds ratio (95% CI)		*P*-value	Adjusted odds ratio (95% CI)		*P*-value
For $MgSO_4$ (with ritodrine)								
Duration of administration	(Missing value: 6)							
No usage	98/1,516	(6.5%)	1			1		
<48 h	19/153	(12.4%)	2.05	(1.22 - 3.46)	0.007	1.72	(0.994 - 2.97)	0.052
2 to 6 days	8/116	(6.9%)	1.07	(0.51 - 2.26)	0.856	0.91	(0.42 - 1.97)	0.816
**7 to 13 days**	14/91	(15.4%)	2.63	(1.44 - 4.82)	0.002	**2.36**	**(1.28 - 4.43)**	**0.006**
14 to 27 days	11/92	(12.0%)	1.97	(1.01 - 3.81)	0.046	1.55	(0.78 - 3.17)	0.208
≥28 days	18/182	(9.9%)	1.59	(0.94 - 2.69)	0.086	1.43	(0.83 - 2.56)	0.185
Maximum rate of injection (mg/hr)	(Missing value: 6)							
No usage	98/1,516	(6.5%)	1			1		
**- 1.19**	31/238	(13.0%)	2.17	(1.41 - 3.33)	<0.001	**1.83**	**(1.17 - 2.86)**	**0.009**
**1.20 - 1.59**	25/191	(13.1%)	2.18	(1.37 - 3.48)	0.001	**2.03**	**(1.24 - 3.33)**	**0.005**
1.60 -	12/205	(5.9%)	0.90	(0.49 - 1.67)	0.737	0.74	(0.38 - 1.44)	0.378
Final rate of injection (mg/hr) just before cessation	(Missing value: 4)							
No usage	98/1,516	(6.5%)	1			1		
**- 1.19**	42/344	(12.2%)	2.01	(1.37 - 2.95)	<0.001	**1.72**	**(1.15 - 2.57)**	**0.008**
**1.20 - 1.59**	24/171	(14.0%)	2.36	(1.47 - 3.81)	<0.001	**2.17**	**(1.30 - 3.62)**	**0.003**
1.60 -	3/121	(2.5%)	0.37	(0.12 - 1.18)	0.092	0.32	(0.10 - 1.05)	0.060
Time (h) from cessation to delivery	(Missing value: 25)							
No usage	98/1,516	(6.5%)	1			1		
**0 - 1.9**	38/324	(11.7%)	1.92	(1.29 - 2.86)	0.001	**1.65**	**(1.08 - 2.53)**	**0.021**
2.0 - 3.9	10/73	(13.7%)	2.30	(1.14 - 4.62)	0.020	1.97	(0.95 - 4.10)	0.068
≥4.0	19/218	(8.7%)	1.38	(0.83 - 2.31)	0.217	1.24	(0.72 - 2.16)	0.441

Abbreviations: $MgSO_4$, magnesium sulfate; CI, confidence interval; h, hour(s); min, minute.

This analysis was performed using the "Hyperkalemia: Both ritodrine and $MgSO_4$ plus control set" derived from "Hyperkalemia set".

a: Multivariable analyses were adjusted by the following 13 variables: obstetrical complications (pPROM, GH/PE/eclampsia/HELLP/AFLP, placental abruption, placenta previa/low-lying placenta, DM, and GDM), cesarean section, delivery at <35 wk, twins/triplets, infantile sex, SGA infants,large-for-gestational-age infants, and Apgar score at 1 min <3. For "duration of administration", "maximum rate of injection", "final rate of injection just before cessation", and "time from the cessation to delivery", excluding 33, 33, 31, and 52 patients with missing data for 13 variables, totals of 2,123, 2,123, 2,125, and 2,104 patients were used for multivariable analysis, respectively. Abbreviations: pPROM, preterm premature rupture of the membranes; GH, gestational hypertension; PE, preeclampsia; HELLP, hemolysis, elevated liver enzymes, and low platelets; AFLP, acute fatty liver of pregnancy; DM, diabetes mellitus; GDM, gestational diabetes mellitus; wk, gestational weeks; SGA, small-for-gestational-age.

Supplementary Table S5 Maternal and infantile characteristics in 4,279 infants with data on cerebral palsy who were born at 32-36 gestational weeks

Characteristics	Non-cerebral palsy (N = 4256)	Cerebral palsy (N = 23)	Missing value	*P*-value
Maternal characteristics				
Age (yr)	33.5 (29.5 - 36.5)	31.5 (27.5 - 37.5)	2/0	0.399
Nulliparity	2158 (50.7)	16 (69.6)	0	0.093
Possible maternal risk factors for cerebral palsy				
Obstetrical complications				
TPL/shortened CL/CI	2452 (57.6)	11 (47.8)	0	0.400
pPROM	1129 (26.5)	6 (26.1)	0	1.000
GH/PE/eclampsia/HELLP/AFLP	656 (15.4)	3 (13.0)	0	1.000
**Placental abruption**	127 (3.0)	3 (13.0)	0	**0.031**
Placenta previa/Low-lying placenta	278 (6.5)	1 (4.3)	0	1.000
DM	45 (1.1)	0 (0)	0	1.000
GDM	235 (5.5)	1 (4.3)	0	1.000
Cesarean section	2698/4212 (64.1)	18/22 (81.8)	45	0.117
$MgSO_4$ usage	916 (21.5)	5 (21.7)	0	1.000
Ritodrine usage	2233 (52.5)	12 (52.2)	0	1.000
Combination of $MgSO_4$ and ritodrine			0	0.991
Neither $MgSO_4$ nor ritodrine	1790 (42.1)	10 (43.5)		
$MgSO_4$ alone	233 (5.5)	1 (4.3)		
Ritodrine alone	1550 (36.4)	8 (34.8)		
Both $MgSO_4$ and ritodrine	683 (16.0)	4 (17.4)		
Possible children's risk factors for cerebral palsy				
**Gestational weeks at delivery**	35.6 (34.2 - 36.4)	33.6 (33.1 - 34.1)	0	**<0.001**
**Delivery at <35 wk**	1613 (37.9)	18 (78.3)	0	**<0.001**
**Birthweight (g)**	2192 (1890 - 2462)	1916 (1752 - 2036)	0	**0.001**
Twins/Triplets	1344 (31.6)	4 (17.4)	0	0.179
Sex: male	2365/4255 (55.6)	14 (60.9)	1	0.678
SGA infants	514 (12.1)	2 (8.7)	0	1.000
Large-for-gestational-age infants	50 (1.2)	0 (0)	0	1.000
**Apgar score at 1 min <3**	112/4252 (2.6)	8 (34.8)	4	**<0.001**
Hypoglycemia at <48 h after birth	1341/4147 (32.3)	11/20 (55.0)	112	0.052
Hyperkalemia at <48 h after birth	257/3413 (7.5)	3 (13.0)	843	0.250

Abbreviations: yr, years old; TPL, threatened preterm labor; CL, cervical length; CI, cervical incompetency; pPROM, preterm premature rupture of the membranes; GH, gestational hypertension; PE, preeclampsia; HELLP, hemolysis, elevated liver enzymes, and low platelets; AFLP, acute fatty liver of pregnancy; DM, diabetes mellitus; GDM, gestational diabetes mellitus; wk, gestational weeks; SGA, small-for-gestational-age; min, minute; h, hour(s).

This analysis was performed using ”Hypoglycemia set”.

Continuous variables are shown as median (interquartile range), and discrete variables are shown as n (%).

The statistical differences between infants with vs. without hypoglycemia were tested using the Mann-Whitney test, Fisher's exact test, or χ^2 test.

Supplementary Table S6 Maternal and infantile characteristics in 4,279 infants with data on any neurological impairments who were born at 32-36 gestational weeks

Characteristics	Non-neurological impairments (N = 4086)	Any neurological impairments (N = 193)	Missing value	*P*-value
Matern+B8:K37al characteristics				
Age (yr)	33.5 (29.5 - 36.5)	32.5 (29.5 - 36.5)	2/0	0.556
**Nulliparity**	2061 (50.4)	113 (58.5)	0	**0.032**
Possible maternal risk factors for cerebral palsy				
Obstetrical complications				
**TPL/shortened CL/CI**	2368 (58.0)	95 (49.2)	0	**0.017**
pPROM	1082 (26.5)	53 (27.5)	0	0.739
**GH/PE/eclampsia/HELLP/AFLP**	618 (15.1)	41 (21.2)	0	**0.025**
**Placental abruption**	114 (2.8)	16 (8.3)	0	**<0.001**
Placenta previa/Low-lying placenta	272 (6.7)	7 (3.6)	0	0.101
DM	43 (1.1)	2 (1.0)	0	1.000
GDM	227 (5.6)	9 (4.7)	0	0.746
Cesarean section	2587/4042 (64.0)	129/192 (67.2)	44/1	0.397
$MgSO_4$ usage	872 (21.3)	49 (25.4)	0	0.180
Ritodrine usage	2143 (52.4)	102 (52.8)	0	0.941
Combination of $MgSO_4$ and ritodrine				
Neither $MgSO_4$ nor ritodrine	1725 (42.2)	75 (38.9)	0	0.308
$MgSO_4$ alone	218 (5.3)	16 (8.3)		
Ritodrine alone	1489 (36.4)	69 (35.8)		
Both $MgSO_4$ and ritodrine	654 (16.0)	33 (17.1)		
Possible children's risk factors for cerebral palsy				
**Gestational weeks at delivery**	35.6 (34.4 - 36.4)	34.1 (33.2 - 35.5)	0	**<0.001**
**Delivery at <35 wk**	1506 (36.9)	125 (64.8)	0	**<0.001**
**Birthweight (g)**	2204 (1906 - 2470)	1900 (1513 - 2184)	0	**<0.001**
**Twins/Triplets**	1301 (31.8)	47 (24.4)	0	**0.032**
**Sex: male**	549/4085 (54.9)	137 (71.0)	1	**<0.001**
**SGA infants**	477 (11.7)	39 (20.2)	0	**<0.001**
Large-for-gestational-age infants	49 (1.2)	1 (4.5)	0	0.727
**Apgar score at 1 min <3**	107/4083 (2.6)	13/192 (6.8)	4	**0.003**
**Hypoglycemia at <48 h after birth**	1275/3983 (32.0)	77/184 (41.8)	112	**0.006**
Hyperkalemia at <48 h after birth	242/3257 (7.4)	18/179 (10.1)	843	0.192

Abbreviations: yr, years old; TPL, threatened preterm labor; CL, cervical length; CI, cervical incompetency; pPROM, preterm premature rupture of the membranes; GH, gestational hypertension; PE, preeclampsia; HELLP, hemolysis, elevated liver enzymes, and low platelets; AFLP, acute fatty liver of pregnancy; DM, diabetes mellitus; GDM, gestational diabetes mellitus; wk, gestational weeks; SGA, small-for-gestational-age; min, minute; h, hour(s).

This analysis was performed using ”Hypoglycemia set”.

Continuous variables are shown as median (interquartile range), and discrete variables are shown as n (%).

The statistical differences between infants with vs. without hypoglycemia were tested using the Mann-Whitney test, Fisher's exact test, or χ^2 test.

Table S7 Effects of various risk factors on the occurrence of any neurological impairments in 4,279 infants who were born at 32-36 gestational weeks

Risk factors[a]	Univariable analysis			Multivariable analysis[b]		
	Crude odds ratio (95% CI)		*P*-value	Adjusted odds ratio (95% CI)		*P*-value
Combination of $MgSO_4$ and ritodrine						
$MgSO_4$ alone vs. no usage	1.69	(0.97 - 2.95)	0.066	1.49	(0.78 - 2.84)	0.224
Ritodrine alone vs. no usage	1.07	(0.76 - 1.50)	0.709	0.97	(0.66 - 1.43)	0.863
Both $MgSO_4$ and ritodrine vs. no usage	1.16	(0.76 - 1.77)	0.486	1.02	(0.64 - 1.65)	0.926
Obstetrical complications						
pPROM	1.05	(0.76 - 1.45)	0.763	0.94	(0.64 - 1.38)	0.744
GH/PE/eclampsia/HELLP/AFLP	1.51	(1.06 - 2.16)	<0.001	0.92	(0.58 - 1.47)	0.733
**Placental abruption**	3.15	(1.83 - 5.43)	<0.001	**2.43**	**(1.29 - 4.58)**	**0.006**
Placenta previa/Low lying placenta	0.53	(0.25 - 1.13)	0.101	0.43	(0.17 - 1.08)	0.073
DM	0.99	(0.24 - 4.09)	0.983	1.21	(0.28 - 5.28)	0.796
GDM	0.83	(0.42 - 1.65)	0.596	0.91	(0.43 - 1.92)	0.795
Cesarean section	1.15	(0.85 - 1.67)	0.369	1.29	(0.87 - 1.91)	0.800
**Delivery at <35 wk**	3.15	(2.33 - 4.26)	<0.001	**2.31**	**(1.64 - 3.26)**	**<0.001**
Twins/triplets	0.69	(0.49 - 0.96)	0.029	0.77	(0.52 - 1.15)	0.201
**Sex: male**	2.01	(1.47 - 2.76)	<0.001	**2.07**	**(1.47 - 2.93)**	**<0.001**
**SGA infants**	1.92	(1.33 - 2.76)	<0.001	**1.72**	**(1.15 - 2.59)**	**0.009**
Large-for-gestational-age infants	0.43	(0.06 - 3.12)	0.404	0.75	(0.10 - 5.72)	0.778
Apgar score at 1 min <3	2.70	(1.49 - 4.89)	0.001	1.65	(0.83 - 3.26)	0.154
**Hypoglycemia at <48 h after birth**	1.53	(1.13 - 2.06)	0.006	**1.51**	**(1.08 - 2.11)**	**0.016**
Hyperkalemia at <48 h after birth	1.39	(0.84 - 2.31)	0.198	1.37	(0.81 - 2.34)	0.242

Abbreviations: CI, confidence interval; $MgSO_4$, magnesium sulfate; pPROM, preterm premature rupture of the membranes; GH, gestational hypertension; PE, preeclampsia; HELLP, hemolysis, elevated liver enzymes, and low platelets; AFLP, acute fatty liver of pregnancy; DM, diabetes mellitus; GDM, gestational diabetes mellitus; wk, gestational weeks; SGA, small-for-gestational-age; min, minute.

This analysis was performed using "Hypoglycemia set".

a: Risk factors were determined based on both clinical relevance and univariable analysis as follows: combination of $MgSO_4$ and ritodrine, obstetrical complications, cesarean section, delivery at <35 wk, twins/triplets, infantile sex, SGA infants,large-for-gestational-age infants, Apgar score at 1 min <3, hypoglycemia at <48 h after birth, and hyperkalemia at <48 h after birth.

b: Multivariable analyses were performed using the same risk factors as in univariate analyses. However, birthweight was not used as a risk factor due to the close relationship with gestational weeks. In addition, the obstetric complication of TPL/shortened CL/CI was not used as a risk factor because either ritodrine or $MgSO_4$ was commonly used under these conditions. Excluding 989 patients with missing data for 16 variables, a total of 3,290 patients underweint multivariable analysis. Abbreviations: TPL, threatened preterm labor; CL, cervical length; CI, cervical incompetency.

Supplementary Table S8 Maternal and infantile characteristics involving 6,136 infants who were used in the current study, and the other 18,807 infants who were not used.

Characteristics	Infants who were used in the current study (N = 6,136)	Iinfants who were not used in the current study (N = 18,807)	Missing data	*P* -value
Maternal characteristics				
Age (yr)	33.5 (29.5 - 36.5)	33.5 (29.5 - 36.5)	2	0.008
Nulliparity	3,101/6,125 (50.6)	9,665/18,761 (51.5)	48	0.227
Obstetrical complications				
PL/shortened CL/CI	3,394 (55.3)	9,432 (50.2)	0	<0.001
pPROM	1,575 (25.7)	4,591 (24.4)	0	0.048
GH/PE/eclampsia/HELLP/AFLP	945 (15.4)	2,733 (14.5)	0	0.097
Placental abruption	211 (3.4)	819 (4.4)	0	0.002
Placenta previa/Low-lying placenta	384 (6.3)	1,283 (6.8)	0	0.127
DM	57 (0.9)	172 (0.9)	0	0.944
GDM	320 (5.2)	975 (5.2)	0	0.923
Cesarean section	3,813/6,080 (62.7)	11,586/18,670 (62.1)	193	0.361
Children's characteristics				
Gestational weeks at delivery	35.8 (34.4 - 36.5)	35.8 (34.5 - 36.5)	0	0.008
Delivery at <35 wk	2,180 (35.5)	6,195 (32.9)	0	<0.001
Birthweight (g)	2,226 (1,908 - 2,502)	2,242 (1,942 - 2,520)	26	0.001
Twins/Triplets	1,792 (29.2)	4,965 (26.4)	0	<0.001
Sex: male	3,415/6,132 (55.7)	10,331/18,778 (55.0)	33	0.359
SGA infants	665 (10.8)	1,595 (8.5)	0	<0.001
Large-for-gestational-age infants	74 (1.2)	205 (1.1)	0	0.445
Apgar score at 1 min <3 (excluding 280 IUFD)	221/6,046 (3.7)	534/18,541 (2.9)	76	0.003

Abbreviations: yr, years old; PL, preterm labor; CL, cervical length; CI, cervical incompetency; GH, gestational hypertension; PE, preeclampsia; HELLP, hemolysis, elevated liver enzymes, and low platelets; AFLP, acute fatty liver of pregnancy; DM, diabetes mellitus; GDM, gestational diabetes mellitus; wk, gestational weeks; SGA, small-for-gestational-age; min, minute; IUFD, intrauterine fetal death.

Continuous variables are shown as median (interquartile range), and discrete variables are shown as n (%).

STROBE Statement—Checklist of items that should be included in reports of ***cohort studies***

	Item No	Recommendation	Reported on Page #
**Title and abstract**	1	(*a*) Indicate the study's design with a commonly used term in the title or the abstract	#1, #3
		(*b*) Provide in the abstract an informative and balanced summary of what was done and what was found	#3
**Introduction**			
Background/rationale	2	Explain the scientific background and rationale for the investigation being reported	#4-6
Objectives	3	State specific objectives, including any prespecified hypotheses	#5-6
**Methods**			
Study design	4	Present key elements of study design early in the paper	#15-17
Setting	5	Describe the setting, locations, and relevant dates, including periods of recruitment, exposure, follow-up, and data collection	#15-17
Participants	6	(*a*) Give the eligibility criteria, and the sources and methods of selection of participants. Describe methods of follow-up	#15-17
		(*b*) For matched studies, give matching criteria and number of exposed and unexposed	Not applicable
Variables	7	Clearly define all outcomes, exposures, predictors, potential confounders, and effect modifiers. Give diagnostic criteria, if applicable	#17-20
Data sources/ measurement	8*	For each variable of interest, give sources of data and details of methods of assessment (measurement). Describe comparability of assessment methods if there is more than one group	#17-20
Bias	9	Describe any efforts to address potential sources of bias	#15-17
Study size	10	Explain how the study size was arrived at	Not applicable because of retrospective observational study
Quantitative variables	11	Explain how quantitative variables were handled in the analyses. If applicable, describe which groupings were chosen and why	#17-20
Statistical methods	12	(*a*) Describe all statistical methods, including those used to control for confounding	#20
		(*b*) Describe any methods used to examine subgroups and interactions	Not applicable
		(*c*) Explain how missing data were addressed	Tables 1, 3, S1, S5, S6, S8
		(*d*) If applicable, explain how loss to follow-up was addressed	Not applicable
		(*e*) Describe any sensitivity analyses	Not applicable
**Results**			

Participants	13*	(a) Report numbers of individuals at each stage of study—eg numbers potentially eligible, examined for eligibility, confirmed eligible, included in the study, completing follow-up, and analysed	Figure, Tables 1, 3, S1, S5, S6, S8
		(b) Give reasons for non-participation at each stage	Figure
		(c) Consider use of a flow diagram	Figure
Descriptive data	14*	(a) Give characteristics of study participants (eg demographic, clinical, social) and information on exposures and potential confounders	#6-10, Tables 1, 3, S1, S5, S6
		(b) Indicate number of participants with missing data for each variable of interest	Tables 1, 3, S1, S5, S6
		(c) Summarise follow-up time (eg, average and total amount)	Not applicable
Outcome data	15*	Report numbers of outcome events or summary measures over time	#6-10, Table 1-4, S1-S8
Main results	16	(*a*) Give unadjusted estimates and, if applicable, confounder-adjusted estimates and their precision (eg, 95% confidence interval). Make clear which confounders were adjusted for and why they were included	#6-8, Table 1-4, S1
		(*b*) Report category boundaries when continuous variables were categorized	Table S2-S4
		(*c*) If relevant, consider translating estimates of relative risk into absolute risk for a meaningful time period	Not applicable
Other analyses	17	Report other analyses done—eg analyses of subgroups and interactions, and sensitivity analyses	#8-10, Table S2-S7
**Discussion**			
Key results	18	Summarise key results with reference to study objectives	#10-11
Limitations	19	Discuss limitations of the study, taking into account sources of potential bias or imprecision. Discuss both direction and magnitude of any potential bias	#14-15
Interpretation	20	Give a cautious overall interpretation of results considering objectives, limitations, multiplicity of analyses, results from similar studies, and other relevant evidence	#11-14
Generalisability	21	Discuss the generalisability (external validity) of the study results	Not applicable
**Other information**			
Funding	22	Give the source of funding and the role of the funders for the present study and, if applicable, for the original study on which the present article is based	#31-32

*Give information separately for exposed and unexposed groups.

Note: An Explanation and Elaboration article discusses each checklist item and gives methodological background and published examples of transparent reporting. The STROBE checklist is best used in conjunction with this article (freely available on the Web sites of PLoS Medicine at http://www.plosmedicine.org/, Annals of Internal Medicine at http://www.annals.org/, and Epidemiology at http://www.epidem.com/). Information on the STROBE Initiative is available at http://www.strobe-statement.org.
